# Single-visible-light performed STORM imaging with activatable photoswitches†

**DOI:** 10.1039/d5sc03224e

**Published:** 2025-07-10

**Authors:** Fanghui Li, Mengqi Li, Yiqi Shi, Xinyun Bian, Ning Lv, Shaomeng Guo, Ying Wang, Weijun Zhao, Wei-Hong Zhu

**Affiliations:** a Key Laboratory for Advanced Materials and Joint International Research Laboratory of Precision Chemistry and Molecular Engineering, Feringa Nobel Prize Scientist Joint Research Center, Shanghai Key Laboratory of Functional Materials Chemistry, Institute of Fine Chemicals, East China University of Science & Technology Shanghai 200237 China whzhu@ecust.edu.cn zhwj@ecust.edu.cn; b Center of Photosensitive Chemicals Engineering, East China University of Science and Technology Shanghai 200237 China

## Abstract

Stochastic optical reconstruction microscopy (STORM) overcomes the diffraction limit of optical imaging, facilitating high-resolution visualization of cellular substructures at the nanoscale. Essential to this technique is the development of fluorescent photoswitches. However, existing photoswitches typically rely on sophisticated dual-beam systems that involve harmful UV-light and lack specific recognition of biomolecules. Here we develop unique intracellular biomolecule-activatable photoswitches tailored for single-visible-light performed STORM imaging. Upon incorporating intramolecular proton transfer (IPT) units into the photochromic diarylethene, the all-visible-light driven photoswitches are established with excellent photoresponsive efficiency, high brightness and fluorescence ON-to-OFF contrast ratio, guaranteeing STORM imaging using a single-visible-light (488 nm) by regulating the activation, excitation and deactivation processes. Furthermore, we functionalized the IPT units with biomolecular recognition motifs, creating photoswitches capable of sensing the expression levels of intracellular biomolecules (like glutathione (GSH) or β-galactosidase (β-Gal)) with super-resolution. Our objective is to engineer single-visible-light driven, biomolecule-activatable photoswitches, which will significantly streamline the STORM technique and expand the applicability of super-resolution imaging for the precise mapping of intracellular substructures.

## Abstract 2

To address dual-beam complexity and STORM photoswitches' lack of biomolecular recognition, we develop biomolecule-activatable photoswitches for single-visible-light STORM. This simplifies imaging and enables nanoscale biomolecule visualization.

## Graphical abstract

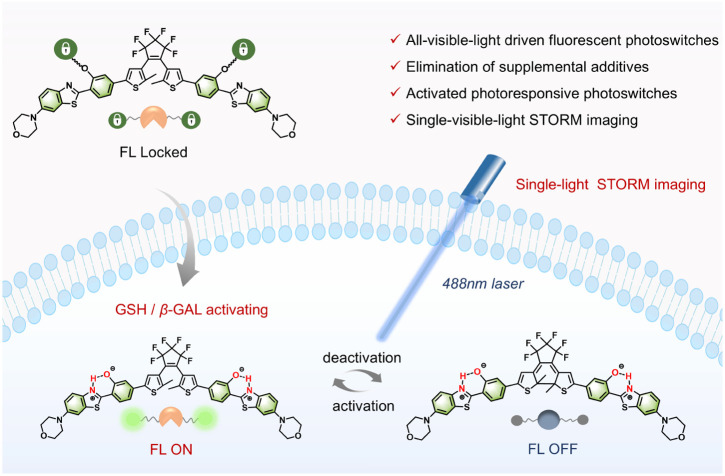

## Introduction

The stochastic optical reconstruction microscopy (STORM) technique overcomes the classical optical diffraction limit,^1,2^ emerging as a powerful super-resolution imaging tool for visualizing biological structures with sub-diffraction spatial resolution.^3,4^ Essential to this technique is the selection of fluorescent photoswitches, whose properties largely determine the quality of STORM images.^5,6^ While existing photoswitch systems have achieved remarkable progress, some limitations persist. For example, reversible photoswitches convert between fluorescent (on) and dark (off) states by two light sources/lasers, which typically involve harmful UV-light,^7–9^ supplemental additives (*e.g.*, oxygen scavenging systems and thiols) are essential for conventional photoswitches (*e.g.*, Cy3B, Alexa Fluor 647) to improve photoresponsive performance and photostability,^10–12^ and the lack of biomolecule-specific responsive photoswitches limits the capability to visualize intracellular biomolecules with high precision.^13,14^ Accordingly, it is desirable to develop novel photoswitches to break through these limitations and broaden the application scope of super-resolution imaging in biological research.

Previous work on STORM systems has produced simpler (single laser wavelength performed STORM) and easier (without additives) protocols,^15–17^ typically involving spontaneous blinking dyes and photochromic dyes as well as quantum dots. Spontaneous blinking dyes^18,19^ (*e.g.*, cyanines, rhodamine derivatives) spontaneously switch between fluorescent and dark states without additional activation of light, but they operate in specific toxic and non-physiological imaging buffers; photochromic dyes, such as turn-on mode fluorescent SDAE,^20^ can use the “hot-band” phenomenon to enable single-visible-light control of activation, excitation and deactivation of fluorescence without additional buffers.^21^ However, due to the extremely low molar extinction coefficients of hot-band absorption, high laser intensities are required for super-resolution imaging; quantum dots^22,23^ have emerged as attractive alternatives through their intrinsic reversible blinking behavior for STORM. Nevertheless, fundamental challenges persist regarding their unclear photoswitching mechanisms and difficulties in functionalization modification.

**Fig. 1:**
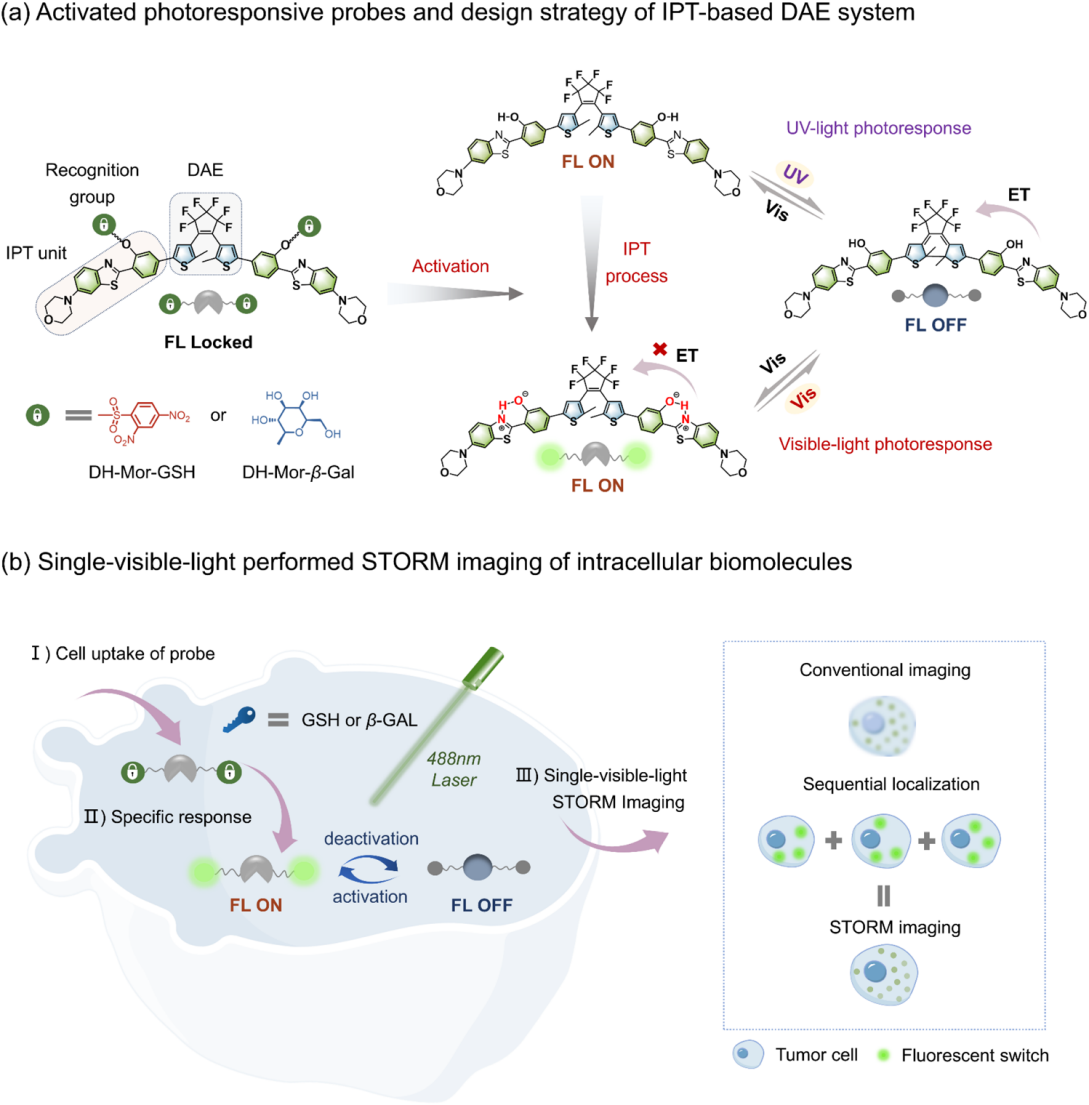
Rational design of unique photoswitchable probes for single-visible-light performed STORM imaging of intracellular biomolecules. (a) Molecular structures of activatable photoswitches and the general design platform of an all-visible-light driven photoresponsive luminescence system employing an intramolecular proton transfer (IPT) strategy. (b) (left): Schematic illustration for single-visible-light performed STORM imaging of GSH or β-Gal with activatable photoswitches. (right): Principle of STORM super-resolution fluorescence microscopy.

Here, we present novel fluorescent photoswitches for single-visible-light driven STORM imaging by incorporating the intramolecular proton transfer (IPT) units of 2-(2-hydroxyphenyl)-benzothiazole (HBT)^24,25^ into photochromic diarylethene (DAE) (Fig. 1a, right). DAE systems,^26–29^ which feature reversible photoinduced fluorescence regulation between the open and closed forms without additional reagents, have attracted increasing attention in super-resolution imaging.^30–35^ Intramolecular proton transfer (IPT) is an important feature of hydrogen-bonded systems (*e.g.*, salicylidene Schiff base and derivatives). The equilibrium between two tautomers OH (proton bonded to oxygen) and NH (proton bonded to nitrogen) determines their optical behavior, with the NH form exhibiting a redshifted absorption band compared to the OH form.^36–38^ Leveraging the distinctive IPT effect, the absorption of the open form is efficiently extended into the visible region, overlapping with that of the closed form, realizing a fully visible-light driven photoresponsive system. Furthermore, a strongly electron-donating morpholine unit was attached to HBT to realize the red-shift of the absorption peak of the open form and the enhancement of the molar extinction coefficient within the visible region. Importantly, the incorporation of the morpholine unit simultaneously improves the fluorescence quantum yield and the visible-light photoresponsive performance of the photoswitch, thus enabling a single 488 nm light performed STORM imaging and greatly simplifying the STORM technique. Finally, we developed two intracellular biomolecule-activatable photoswitches, DH-Mor-GSH and DH-Mor-β-Gal (Fig. 1a, left), by integrating specific recognition motifs to modulate the IPT switching effect. These rationally designed photoswitches exhibit specific responsiveness toward glutathione (GSH) or β-galactosidase (β-Gal), enabling high-resolution visualization of endogenous biomolecules (GSH or β-Gal) (Fig. 1b). Given these activatable photoswitches, we employed the STORM single-molecule counting method to analyze the expression levels of GSH and β-Gal in various cancer cells, thus offering a convenient and intuitive approach for the semiquantitative analysis of intracellular biomolecules. In summary, we offer single-visible-light driven and biomolecule-activatable photoswitches for broadening STORM super-resolution imaging for visualizing intracellular substructures.

## Results and discussion

### All-visible-light driven photoswitches *via* the IPT effect

**Fig. 2:**
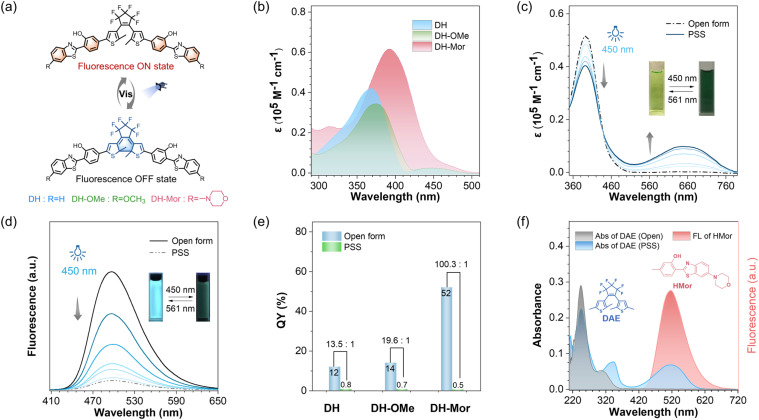
All-visible-light modulated photoresponse and fluorescence behavior of IPT-based DAE. (a) Molecular structure of DHs: DH, DH-OMe and DH-Mor. (b) Absorbance spectra of DH, DH-OMe and DH-Mor in DMSO solution. (c) Absorption spectra changes of DH-Mor upon irradiation with visible light (*λ* = 450 nm). The inset images show the color changes. (d) Fluorescence spectra changes of DH-Mor upon irradiation with visible light (*λ* = 450 nm). The inset images show the fluorescence changes. (e) Fluorescence quantum yields of the open form and PSS (photostability state) of DH, DH-OMe and DH-Mor in toluene solution. (f) The overlap absorbance spectra of the DAE fragment, and the emission spectra of the HMor fragment. The molecular structure of DAE and HMor is shown. The emission spectra of HMor efficiently overlaps the absorption of DAE's closed form.

Since the progress in developing photochromic DAE dyes for STORM imaging is hindered by the requirement for harmful UV-light to induce photocyclization,^39–41^ we rationally designed a series of all-visible-light modulated fluorescent photoswitches, namely DH, DH-OMe and DH-Mor (synthetic methods are described in the ESI†). These novel photoswitches feature the fluorophore HBT which has an IPT effect, and methoxy and morpholine units with varying electron-donating ability to modulate their photoresponsive and fluorescent performance (Fig. 2a).

As anticipated, the open forms of the three designed photoswitches (DH, DH-OMe and DH-Mor) exhibited an additional absorption band in the visible-light region (Fig. 2b). Compared to DH and DH-OMe, DH-Mor with a stronger electron-donating morpholine group showed a more red-shifted absorption band and a significantly higher molar extinction coefficient in the visible-light range (400–500 nm), reaching 0.8 × 10^4^ M^−1^ cm^−1^ at 450 nm. Extending π-conjugation in DAEs is a common strategy to red-shift the absorption band into the visible region,^42–44^ but often leads to a significant reduction in photoresponsive efficiency. With the introduction of the IPT strategy, the three designed photoswitches maintain excellent photoresponsive behavior upon all-visible-light irradiation and show excellent fatigue resistance (Fig. 2c and S1–S3†). The photocyclization quantum yields (*Φ*_o–c_) at 450 nm were measured to be 36% for DH-Mor, accompanied by an excellent photocyclization conversion ratio as high as 92%. Desirable photoswitching efficiencies were also achieved for both DH and DH-OMe upon visible light irradiation at 450 nm (Table 1). Notably, the photocyclization quantum yields measured at 450 nm were lower than those obtained under 365 nm irradiation. This decrease is attributed to enhanced competitive photocycloreversion reactions at the longer wavelength. Surprisingly, DH-Mor exhibited both the highest photocyclization quantum yield and conversion ratio under 450 nm light irradiation, with a photocyclization rate approximately 47 times higher than that of the DH-OMe (Fig. S7†). In summary, the rational design of IPT-based DAE realizes desirable all-visible-light driven photoresponsive performance, and DH-Mor with the strong electron-donating morpholine group shows the highest photoresponsive efficiency.

**Table 1:** Photochromic data of DHs

Compound^a^	Photocyclization			Photocycloreversion		
	*λ*_irr_ b [nm]	CR_o–c_^c^ [%]	*Φ*_o–c_ c [%]	*λ*_irr_ b [nm]	CR_c–o_^d^ [%]	*Φ*_c–o_ d [%]
DH	365	93	37	600	>99	7.6
	450	62	27			
DH-OMe	365	98	40	600	>99	5.5
	450	69	29			
DH-Mor	365	>99	42	600	>99	2.3
	450	92	36			

a Solvent for DHs: DMSO.

b *λ*_irr_ represents the wavelength of light for irradiation.

c CR_o–c_ and *Φ*_o–c_ represent the conversion ratio and quantum yield of photocyclization, respectively.

d CR_c–o_ and *Φ*_c–o_ represent the conversion ratio and quantum yield of photocycloreversion, respectively.

### Efficient fluorescence modulation *via* a morpholine moiety

In addition to photoresponsive performance, the fluorescence characteristics, such as fluorescence brightness and the ability to modulate between fluorescence ON and OFF states, are critical factors for fluorescent photoswitches.^45,46^ Notably, the open form of DH-Mor exhibited brightest green fluorescence, with a fluorescence quantum yield (*Φ*_F_) of 52%, approximately four times higher than those of DH and DH-OMe. Upon dual visible-light (450 and 561 nm) irradiation, the green fluorescence of DH-Mor was gradually and reversibly switched, with a fluorescence ON-to-OFF contrast ratio of 100.3, significantly higher than those of DH (13.6) and DH-OMe (19.5) (Fig. 2d and e). DH-Mor is a classical turn-off mode fluorescent molecule: the open form is fluorescent, while the closed form remains in a dark state. This efficient fluorescence conversion can be attributed to the intermolecular energy transfer process between the fluorescent IPT unit and the photochromic DAE core (Fig. 2f). Overall, through donor molecular engineering, the fluorescence photoswitch DH-Mor exhibited both the highest brightness and fluorescence ON-to-OFF contrast ratio, making it highly promising for STORM imaging.

### Biomolecule-activated fluorescent photoswitches

**Fig. 3:**
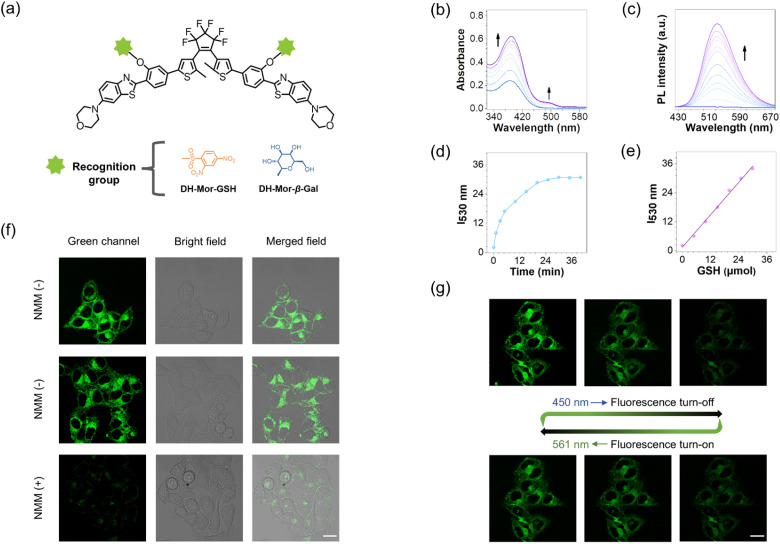
Construction of activatable fluorescent photoswitches. (a) Molecular structures of DH-Mor-GSH and DH-Mor-β-Gal. (b) Absorbance and (c) fluorescence spectra of DH-Mor-GSH (10 μM) incubated with GSH in a mixture solution of PBS/DMSO solution (1 : 9, v/v) at 37 °C. (d) Time-dependent fluorescence intensity at 530 nm (0–35 min) for DH-Mor-GSH incubated with GSH. (e) Emission changes plots of fluorescence intensity at 530 nm for DH-Mor-GSH as a function of GSH concentration (0–30 μM). (f) Confocal imaging of HeLa cells incubated with DH-Mor-GSH. NMM (−) represents the live cells are only incubated with 10 μM DH-Mor-GSH for 30 min, and NMM (+) represents the live cells are pretreated with a derivative of covalently sequestered GSH methylmaleimide (NMM) (500 μM) for 30 minutes to deplete cellular GSH, and then incubated with DH-Mor-GSH. Scale bar, 20 μm. (g) Fluorescence turn-off and turn-on behaviors in fixed HeLa cells recorded by confocal laser scanning microscopy. Scale bar, 20 μm.

Glutathione (GSH) and β-galactosidase (β-Gal)^47–51^ are vital biomolecules in living organisms, crucial for cellular physiological processes. However, the nano-level visualization of intracellular GSH or β-Gal remains challenging due to the limited resolution of traditional microscopes and the lack of responsive photoswitches.^52–54^ Given the high-performance photoswitch DH-Mor, we constructed activatable photoswitches DH-Mor-GSH and DH-Mor-β-Gal by incorporating GSH or β-Gal recognition motifs into the phenolic hydroxyl reactive oxygen site within the IPT motif (Fig. 3a). Prior to cellular experiments, we also validated the performance of DH-Mor under physiologically relevant aqueous conditions (PBS) to ensure intracellular functionality. As shown in Fig. S8,† DH-Mor maintains all-visible-light driven photoresponsive performance and good fatigue resistance in aqueous media. As illustrated in Fig. 3b–d, both the absorbance and fluorescence of DH-Mor-GSH rapidly responded to GSH, reaching equilibrium within 30 min at physiological temperatures (37 °C). The fluorescence intensity of DH-Mor-GSH had a positive linear relationship to GSH concentration (0–30 μM) (Fig. 3e), indicating the potential for quantitative detection of endogenous GSH. Notably, DH-Mor-GSH retained excellent all-visible-light modulated photoresponsive performance when reacting with GSH (Fig. S9†). Similarly, DH-Mor-β-Gal demonstrated a promising activatable response towards β-Gal *in vitro* (Fig. S11†).

To validate the effectiveness of DH-Mor-GSH in detecting GSH and the photoresponsive properties within the cells, intracellular confocal imaging experiments were first conducted. The photoswitches showed low cytotoxicity in living cells (Fig. S12†), making them suitable for intracellular imaging. Upon treatment with DH-Mor-GSH, HeLa cells exhibited intense fluorescence (Fig. 3f), in contrast with the very weak fluorescence in cells pretreated with NEM (a well-known thiol scavenger), confirming the specific responsiveness of DH-Mor-GSH to endogenous GSH. Meanwhile, the reversible modulation of intracellular fluorescence with alternating irradiation at 450 and 561 nm demonstrated excellent photoresponsive properties within the cells (Fig. 3g). The β-Gal activatable photoswitch DH-Mor-β-Gal also exhibited outstanding recognition ability for endogenous β-Gal (Fig. S13 and S14†).

### Single-visible-light performed STORM imaging

Conventional STORM technology typically requires two light sources to reversibly convert photoswitches between fluorescence ON and OFF states. Our photoswitch DH-Mor has an effective overlap of the absorption spectra of its open and closed forms in the visible range, making it highly capable of modulating fluorescence with a single light source.

**Fig. 4:**
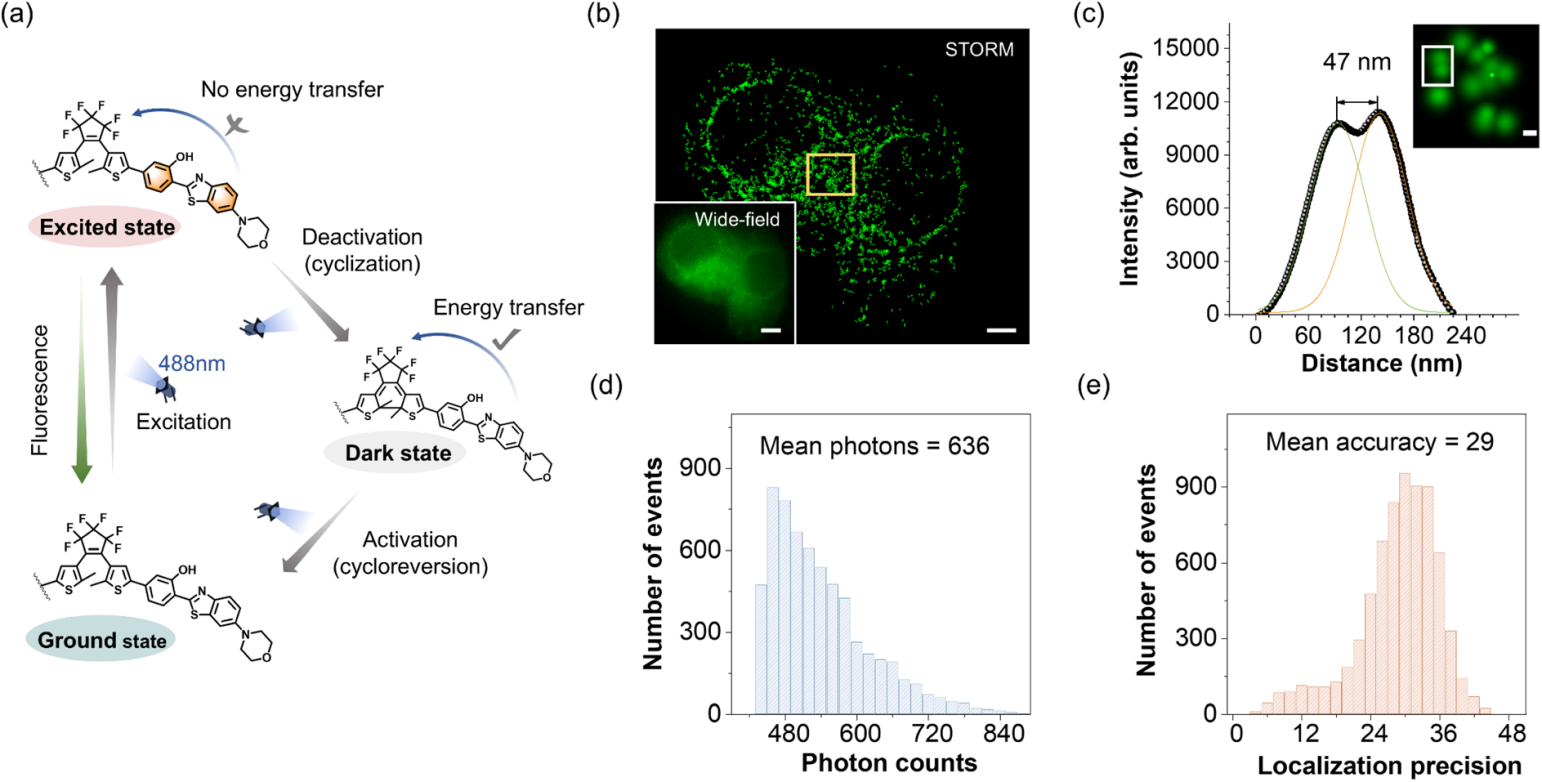
Single-visible-light performed STORM imaging of intracellular biomolecules. (a) Mechanism of 488 nm single light modulation of activation, excitation and deactivation processes of the photoswitch. (b) STORM and conventional wide-field images of a HeLa cell. STORM image, scale bar, 4 μm. Wide-field image, scale bar, 5 μm. (c) Fluorescence intensity cross-sectional profiles of two pairs of adjacent molecules in the yellow square region for (b). Scale bar, 0.1 μm. (d) Photon counts distribution at each localization event in STORM imaging of (b). (e) Localization accuracy distribution at each localization event in STORM imaging of (b).

Here we propose a mechanism illustration for STORM imaging using a single 488 nm laser (spectral adaptation to DH-Mor) (Fig. 4a): (1) exciting the probe's fluorescence, (2) switching off the fluorescence by inducing cyclization to its fluorescence OFF state, (3) activating the photoswitch to its fluorescence ON state *via* cycloreversion. To validate this mechanism, we conducted confocal imaging using a 488 nm laser on DH-Mor labeled micelles (formed from PSt_38k_-*b*-PEO_11k_ copolymer self-assembly). Initially, the fluorescence of the open form of DH-Mor molecules was excited by a 488 nm laser, the micelles exhibit intense fluorescence, which slightly decreases upon irradiation and eventually reaches a photostationary state. Initial fluorescence (100% open form) decreased to 92% at the PSS state (Fig. S15†). This imaging process clearly reflects the dynamic fluorescence switching of DH-Mor driven by a single 488 nm laser. In detail, the 488 nm laser excites each DH-Mor molecule to emit photons and enter the fluorescence OFF state, while simultaneously reactivating it to return to the fluorescence ON state, enabling reversible switching between fluorescence ON and OFF states. By employing a single 488 nm laser, we observed distinct fluorescent blinking behavior within the micelles. The STORM imaging of DH-Mor labeled micelles significantly enhances image resolution with a full-width at half-maximum (FWHM) of 47 nm, a ninefold reduction compared to conventional widefield fluorescence imaging (Fig. S16†). Moreover, the spatial resolution was analyzed using Fourier ring correlation (FRC),^55–57^ a method recently often used to determine the resolution of localization-based super-resolution fluorescence imaging. The resolution of the localizations was determined to be about 63 nm (Fig. S17†).

We further conducted STORM imaging using intracellular biomolecule-activatable photoswitches, DH-Mor-GSH and DH-Mor-β-Gal, with a single laser beam. Upon intracellular GSH activation, we performed STORM imaging using a single 488 nm laser. The super-resolution imaging exhibited a significant improvement in spatial resolution compared to conventional wide-field and confocal microscopy. As shown in Fig. 4b and S18,† traditional imaging approaches demonstrated limited capacity to resolve the nanoscale spatial distribution of intracellular GSH due to their inherent diffraction-limited resolution constraints. In contrast, STORM imaging successfully resolved intracellular GSH localization with a measured overall spatial resolution of 69 nm (Fig. S20†) as measured by FRC. Notably, STORM image exhibited a clear distinction between two localized points separated by a distance as small as 47 nm (Fig. 4c). The STORM image exhibited an average photon count (636 per switch) and average localization precision (29 per switch) (Fig. 4d and e), enabling the localization and visualization of endogenous GSH with high precision. Super-resolution imaging has clearly revealed the distribution characteristics of GSH within HeLa cells. GSH is predominantly enriched in the cytoplasm, where it participates in a variety of critical biochemical reactions, sustaining the normal physiological activities of the cell.^58^ Upon activation by β-Gal, the activatable photoswitch DH-Mor-β-Gal also realized a single-light performed STORM imaging (Fig. S19†).

### STORM single-molecule counting mode for analyzing the biomolecules' expression levels

**Fig. 5:**
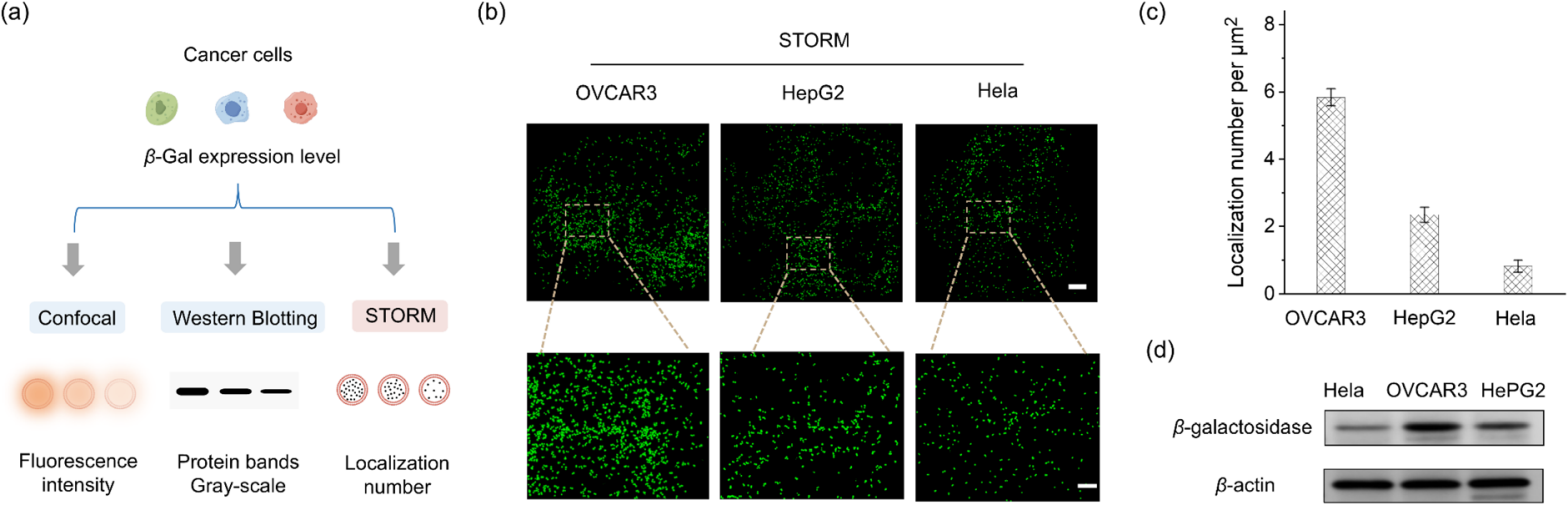
Analyzing the expression levels of β-Gal in different types of cancer cells. (a) Schematic illustration of confocal imaging, western blotting and STORM imaging to analyze the expression levels of endogenous β-Gal in different cancer cells. (b) STORM images of the endogenous β-Gal level in HeLa, HepG2 and OVCAR3 cells determined by DH-Mor-β-Gal (20 μM) staining. Scale bars, 4 μm. Scale of enlarged area, 2 μm. (c) The number of β-Gal localizations per μm^2^ on cells as represented in panel (b). Error bars represent standard deviation, *n* = 3. (d) Western blotting of β-Gal in HeLa, HepG2 and OVCAR3 cells.

STORM technology provides precise localization of single-molecules^59,60^ when adopting our activatable photoswitch capable of specific biomolecule recognition; it becomes an ideal tool for directly analyzing intracellular biomolecule expression levels by quantifying the number of localized molecules in STORM images. As reported, β-Gal expression levels varied across different cancer cells and were typically evaluated using two methods: confocal imaging (based on fluorescence intensity) and western blotting (based on protein bands gray-scale analysis) (Fig. 5a). Using the activatable photoswitch DH-Mor-β-Gal, STORM imaging can clearly demonstrate the distribution of intracellular β-Gal in all three types of cancer cells (Fig. 5b). The counting mode of STORM imaging can provide the localization number per μm^2^, ranked from highest to lowest as follows: OVCAR3, HepG2, and HeLa cells (Fig. 5c), consistent with the trends observed in confocal imaging (Fig. S21†) and western blotting experiments (Fig. 5d), thereby validating the accuracy and convenience of the STORM-based counting method. Moreover, using the STORM single-molecule counting mode, the differentiated expression levels of GSH in HeLa, HepG2, and OVCAR3 cells can be evaluated by activatable probe DH-Mor-GSH (Fig. S22 and S23†), showing the GSH expression levels ranked from highest to lowest as follows: HeLa cells, HepG2 and OVCAR3. These results suggest that the STORM-based single-molecule counting method offers a simpler, more intuitive approach to semiquantitative analysis of intracellular biomolecules, providing a powerful alternative with improved resolution.

## Conclusions

In summary, we have successfully developed novel biomolecule-activatable photoswitches, specifically driven by single-visible-light, eliminating the need for harmful UV-light and supplemental additives. Diverging from conventional methodologies that achieve all-visible-light-regulated diarylethene through extending π-conjugation and utilizing the “hot-band” phenomena, our innovative strategy is to ingeniously integrate morpholine and IPT units into the photoswitch DAE. On the one hand, introducing IPT units generates a strong emerged absorption band in the visible-light region, under 450 nm irradiation, while DH-Mor exhibits a high photocyclization quantum yield (36%) and conversion ratio (92%). On the other hand, the well-chosen morpholine groups endow DH-Mor with a high fluorescence quantum yield (52%) and an enhanced fluorescence ON-to-OFF contrast ratio, ensuring effective fluorescence modulation. These two design strategies synergistically contribute to single-visible-light performed STORM imaging, along with the following advantages: favorable cell permeability and biocompatibility, as well as the elimination of supplemental additives during STORM data acquisition. Furthermore, recognition units for vital biomolecules, such as GSH and β-Gal, are incorporated to switch the IPT effect, yielding unique biomolecule-activatable photoswitches that enable localization of intracellular GSH and β-Gal with high precision. Given these single-visible-light driven and activatable photoswitches, we can visualize expression levels of GSH and β-Gal in various cancer cells using STORM single-molecule counting methods, offering a convenient and precise approach for the semiquantitative analysis of intracellular biomolecules. This innovative design strategy not only simplifies the STORM imaging process but also expands the application scope of super-resolution imaging in biological research.

## Author contributions

W. Zhu and W. Zhao supervised the study and directed the scientific research, and prepared the manuscript. F. L., Y. S., N. L., Y. W. and S. G. carried out the design, synthesis, and optical determination. F. L. and M. L. did the data analysis and wrote the manuscript. F. L. and X. B. performed the bioimaging experiments and analyzed the data.

## Conflicts of interest

There are no conflicts to declare.

## Supplementary Material

SC-016-D5SC03224E-s001

## Data Availability

All relevant data is presented in the paper and ESI.†
