# Supplementary material for: Single-visible-light performed STORM imaging with activatable photoswitches
Source: Chem Sci. 2025 Jul 10;16(31):14270–7. doi: 10.1039/d5sc03224e (PMC12242835; doi:10.1039/d5sc03224e)
Supplement: SC-016-D5SC03224E-s001 [file SC-016-D5SC03224E-s001.pdf]

Supplementary Information (SI) for Chemical Science.
This journal is © The Royal Society of Chemistry 2025

Electronic Supplementary Information (ESI)

Single-Visible-Light Performed STORM Imaging with Activatable Photoswitches

Fanghui Li, ‡[a] Mengqi Li, ‡[a] Yiqi Shi,[a] Xinyun Bian,[a] Ning Lv,[a] Shaomeng Guo,[a] Ying Wang,[a] Weijun Zhao,[a,b]* and Wei-Hong Zhu[a,b]*

[a] Key Laboratory for Advanced Materials and Joint International Research Laboratory of Precision Chemistry and Molecular Engineering, Feringa Nobel Prize Scientist Joint Research Center, Shanghai Key Laboratory of Functional Materials Chemistry, Institute of Fine Chemicals, East China University of Science & Technology, Shanghai 200237, China, E-mail: whzhu@ecust.edu.cn; zhwj@ecust.edu.cn

[b] Center of Photosensitive Chemicals Engineering, East China University of Science and Technology, Shanghai 200237, China.

‡ These authors contributed equally to this work.

Table of Contents

1. Experimental Procedures S3
 1.1 Reagents S3
 1.2 Instruments S3
 1.3 Synthetic route of compounds S4
 1.4 Cell lines S13
 1.5 Western blot S13
 1.6 In vitro cytotoxicity assay S13
 1.7 Confocal laser scanning microscopy S13
 1.8 STORM imaging protocol S14
 1.9 Data analysis S14
 1.10 The preparation of PSt-b-PEO block copolymer micelles S15
 1.11 The fluorescence quantum yield measurement S15
 1.12 The photoisomerization quantum yield measurement S15
 1.13 The fatigue resistance measurement S17
2. Supporting Figures S18
3. Characterization of compounds S29
4. References S43

1. Experimental Procedures

1.1 Reagents

All starting reagents were commercially available and analytical purity, which were used without further treatment. Bis(pinacolato)diboron and $Pd(PPh_3)_4$ were purchased from Adamas Reagent Co., Ltd., 4-morpholinoaniline, 2-amino-6-methoxybenzothiazole and 2-benzothiazolamine were purchased from Shanghai Mairuier Biochemical Technology Co., Ltd.(China), 4-bromo-2-hydroxybenzaldehyde and 2,4-dinitrobenzenesulfonyl chloride were purchased from Aladdin Reagent Co., Ltd., 2,3,4,6-tetra-o-acetyl-alpha-d-galactopyranosyl bromide was purchased from Bidepharm Technology Co., Ltd., perfluorocyclopentene was purchased from J&K Scientific Ltd. TLC (Thin-layer chromatography) analysis was performed on silica-gel plates, and column chromatography was conducted using silica-gel column packages purchased from Qingdao Haiyang Chemical (China). The human hepatocellular carcinomas (HepG2), human ovarian cancer cells (OVCAR3), and human epithelioid cervical carcinoma cell (HeLa) were purchased from the Shanghai Fuheng Biotechnology Co., Ltd (China). Cells were all propagated in T-75 flasks cultured at 37 °C under a humidified 5% CO_2 atmosphere in Dulbecco's modified Eagle's medium (DMEM) (GIBCO/Invitrogen, Camarillo, CA), which was supplemented with 10% fetal bovine serum (FBS; Biological Industry, Kibbutz Beit Haemek, Israel) and 1% penicillin–streptomycin (10,000 U mL^{-1} penicillin and 10 mg mL^{-1} streptomycin; Solarbio Life Science, Beijing, China).

1.2 Instruments

NMR spectra were recorded on Bruker AM-400 spectrometers with tetramethylsilane (TMS) as internal reference, $CDCl_3$, DMSO-d_6 as solvents. High resolution mass (HRMS) spectra were recorded on Waters LCT Premier XE spectrometer. UV/Vis spectra were recorded on Varian Carry 500 (1 cm quartz cell) at 293 K. Fluorescence spectra were recorded using HORIBA Fluoromax 4 at 293 K. The photochromic reaction was induced in situ by continuous irradiation using an Hg/Xe lamp (Hamamatsu, LC8 Lightningcure, 200 W) equipped with a narrow band interference filter (Shenyang HB optical Technology) for λ_{irr} = 546 nm, λ_{irr} = 365 nm and λ_{irr} = 450 nm. Confocal fluorescence images were performed on Lecia TCS SP8 laser scanning confocal microscopy. STORM images were performed on Nikon N-STORM Super Resolution Microscopic Imaging System (100 * TIRF oil mirror, 488nm laser)

1.3 Synthetic route of compounds

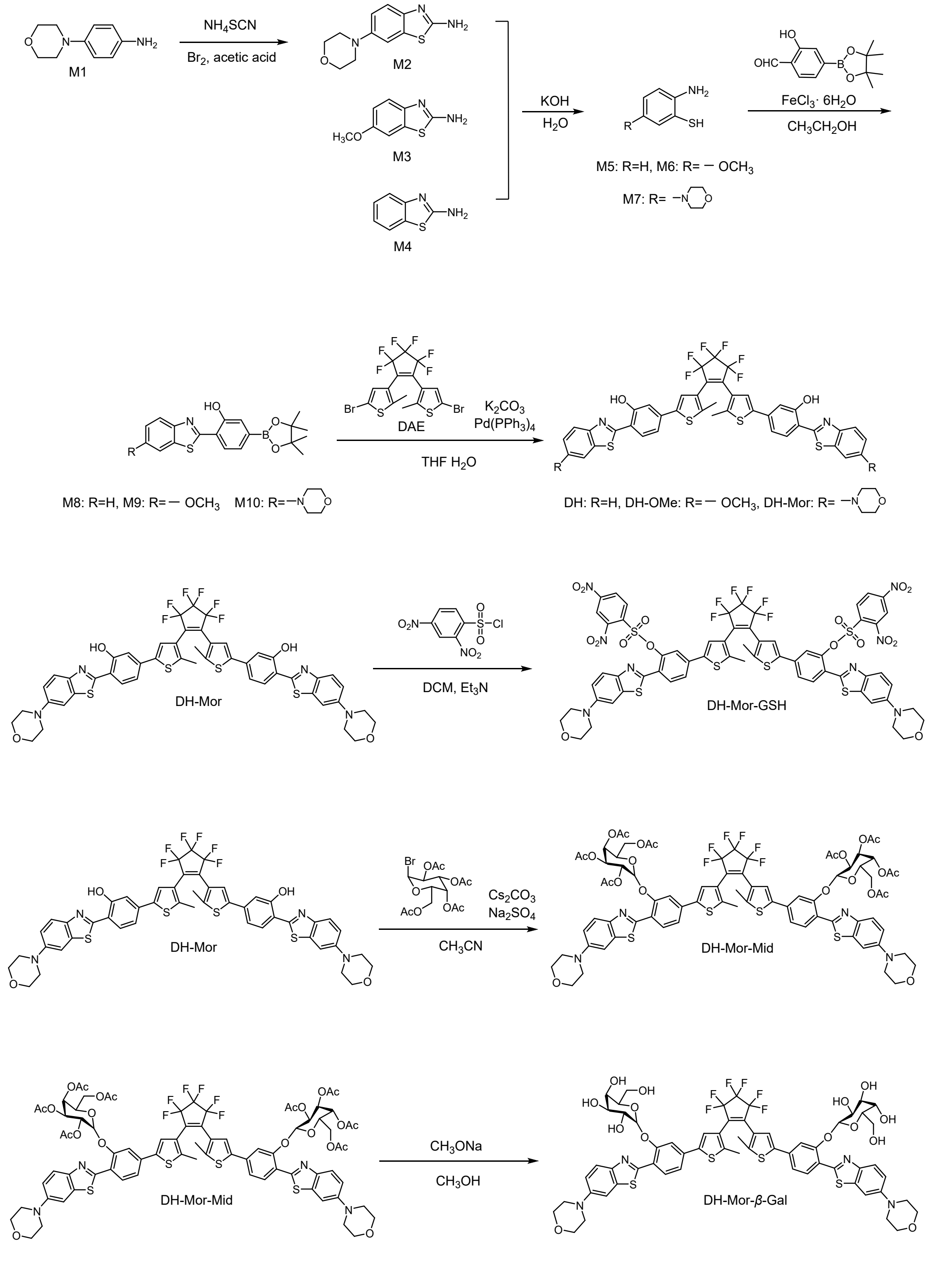

Scheme S1. Synthetic routes of target system.

Synthesis and characterization of M2

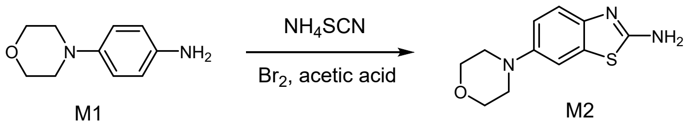

Scheme S2. Synthesis route of **M2**

To a 250 mL single necked bottle, M1 (10.0 g, 56.2 mmol, 1 eq), NH_4SCN (21.4 g, 280.0 mmol, 5 eq), and acetic acid (100 mL) were added. The mixture was cooled to 10 °C and stirred for 20 min, and then bromine (8.9 g, 56.2 mmol, 1 eq) was added drop wise at such a rate to keep the below 10 °C throughout the addition. The reaction mixture was stirred overnight at room temperature, and then poured into hot water, and basified to pH 11 with ammonia solution (NH_4OH). The resulting precipitate was filtered, washed with water, dried and recrystallized from toluene in brown solid **M2** 3.9 g (yield: 30.3%). ^{1}H NMR (400 MHz, DMSO-d_6, ppm), δ: 6.86 (d, J = 6.4 Hz, 1 H, Ar-H), 6.69 (d, J = 8.4 Hz, 1 H, Ar-H), 6.51 (s, 1 H, Ar-H), 5.05 (s, 1 H, Ar-NH_2), 3.65 (t, J = 4.4, 4 H, morpholine-CH_2-CH_2), 2.76 (t, J = 4.4, 4 H, morpholine-CH_2-CH_2). ^{13}C NMR (151 MHz, DMSO-d_6, ppm), δ: 164.958, 146.909, 146.831, 132.472, 118.310, 115.379, 108.396, 66.687, 50.424.

Synthesis and characterization of M5

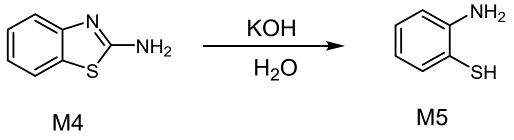

Scheme S3. Synthesis route of **M5**

To a 250 mL single necked bottle, **M4** (5.0 g, 33.1 mmol, 1 eq), KOH (18.5 g, 330.0 mmol, 10 eq) and H_2O (100 mL) were added. The mixture was stirred at 120 °C overnight. After removing the scraps by filtering, the filtrate was neutralized by acetic acid (30% in water), and the precipitate was collected by filtration to give **M5** brown solid 3.7 g (yield: 88.4%). ^{1}H NMR (400 MHz, $CDCl_3$, ppm), δ: 7.28 (dd, J_1 = 7.6, J_2 = 1.6 Hz, 1 H, Ar-H), 7.02 (ddd, J_1 = 8.0, J_2 = 7.4, J_3 = 1.6 Hz, 1 H, Ar-H), 6.57-6.70 (m, 2 H, Ar-H), 4.09 (s, 2 H, Ar-NH_2), 2.85 (s, 1 H, Ar-SH). ^{13}C NMR (151 MHz, DMSO-d_6, ppm), δ: 150.214, 135.884, 131.607, 116.937, 116.535, 115.265.

Synthesis and characterization of M6

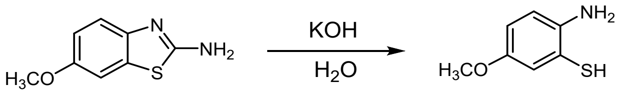

M3 M6

Scheme S4. Synthesis route of **M6**

To a 250 mL single necked bottle, **M3** (5.0 g, 28.3 mmol, 1 eq), KOH (15.7 g, 280.1 mmol, 10 eq) and H_2O (100 mL) were added. The mixture was stirred at 120 °C overnight. After removing the scraps by filtering, the filtrate was neutralized by acetic acid (30% in water), and the precipitate was collected by filtration to give **M6** brown solid 3.2g (yield: 75.3%). ^1H NMR (400 MHz, DMSO-d_6, ppm), δ: 6.77 (dd, J_1 =8.8 Hz, J_2 =2.8 Hz, 1 H, Ar-H), 6.70 (d, J = 8.0, 1 H, Ar-H), 6.59 (d, J = 2.8 Hz, 1 H, Ar-H), 5.04(s, 2 H, Ar-NH_2), 3.53 (s, 3 H, -O-CH_3). ^{13}C NMR (151 MHz, DMSO-d_6, ppm), δ: 150.572, 144.152, 119.088, 118.992, 117.525, 116.600, 55.732.

Synthesis and characterization of M7

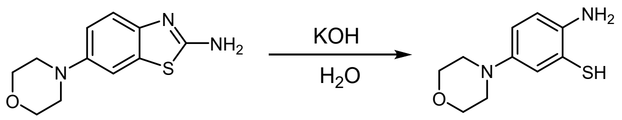

M2 M7

Scheme S5. Synthesis route of **M7**

To a 250 mL single necked bottle, **M2** (5.0 g, 21.3 mmol, 1 eq), KOH (11.7 g, 210.3 mmol, 10 eq) and H_2O (100 mL) were added. The mixture was stirred at 120 °C overnight. After removing the scraps by filtering, the filtrate was neutralized by acetic acid (30% in water), and the precipitate was collected by filtration to give **M7** yellow solid 2.7 g (yield 60.3%). ^1H NMR(400 MHz, DMSO-d_6, ppm), δ: 6.86 (dd, J_1 = 8.8 Hz, J_2 = 2.8 Hz, 1 H, Ar-H), 6.69 (d, J = 8.8 Hz, 1 H, Ar-H), 6.52 (d, J = 2.6 Hz, 1 H, Ar-H), 5.04 (s, 2 H, Ar-NH_2), 3.65 (t, J = 8.0, 4 H, morpholine-CH_2.CH_2), 2.77 (t, J = 8.0, 8 H, morpholine-CH_2-CH_2). ^{13}C NMR (151 MHz, DMSO-d_6, ppm), δ: 143.898, 142.689, 122.837, 121.527, 117.434, 116.439, 116.404, 66.654, 50.669.

Synthesis and characterization of M8

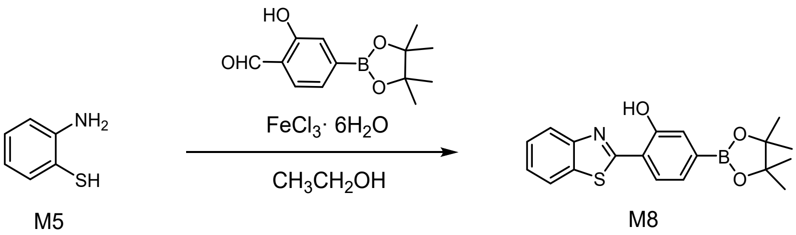

Scheme S6. Synthesis route of **M8**

To a 250 mL single necked bottle, **M5** (5.0 g, 40.0 mmol, 1 eq), 4-formyl-3-hydroxyphenylboronic acid pinacol ester (6.2 g, 25.2 mmol, 1.2 eq) and ethanol (100 mL) were added. The mixture was heated to 95 °C for 4 h. The organic solvents were removed by vacuum rotary evaporation, the precipitate was dissolved in THF solution, and was added dropwise into $FeCl_3 \cdot 6H_2O$ (22.7 g, 84.0 mmol, 4 eq) dissolved in ethanol, the mixture was heated to 80 °C, then the organic layer was concentrated in vacuum. The residue was extracted with dichloromethane (50 mL × 3), The organic layer was separated and dried by anhydrous Na_2SO_4. After concentrated in vacuum, the residue was purified by column chromatography on silica gel (PE: EA = 10:1). A yellow solid **M8** 6.4 g was obtained in 43.8% yield. ^{1}H NMR (400 MHz, $CDCl_3$, ppm), δ: 12.39 (s, 1 H, -OH), 8.33 (d, J = 8.0 Hz, 1 H, Ar-H), 8.25 (d, J = 7.6 Hz, 1 H, Ar-H), 8.03 (d, J = 7.6 Hz, 1 H, Ar-H), 7.89 (m, 1 H, Ar-H), 7.85 (t, J = 7.2 Hz, 1 H, Ar-H), 7.75 (t, J = 7.2 Hz, 1 H, Ar-H), 7.70 (d, J = 7.6 Hz, 1 H, Ar-H), 1.71 (s, 12 H, $-CH_3$). ^{13}C NMR (151 MHz, $CDCl_3$, ppm), δ: 169.283, 157.034, 151.790, 132.886, 127.561, 126.753, 125.681, 125.298, 124.194, 122.305, 121.555, 118.817, 84.152, 24.875.

Synthesis and characterization of M9

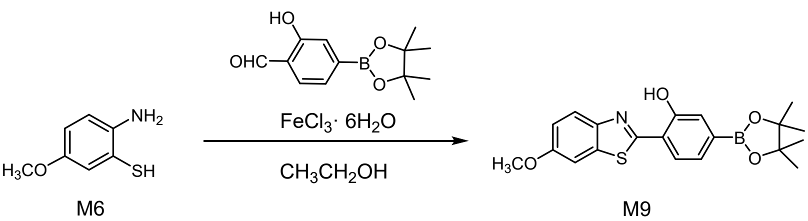

Scheme S7. Synthesis route of **M9**

To a 250 mL single necked bottle, **M6** (5.0 g, 32.3 mmol, 1 eq), 4-formyl-3-hydroxyphenylboronic acid pinacol ester (9.6 g, 38.7 mmol, 1.2 eq) and ethanol (100 mL) were added. The mixture was heated to 95 °C for 4 h. The organic solvents were removed by vacuum rotary evaporation, the precipitate

was dissolved in THF solution, and was added dropwise into $FeCl_3 \cdot 6H_2O$ (32.3 g, 129.2 mmol, 4 eq) dissolved in ethanol, the mixture was heated at 80 °C for 2 h, then the organic layer was concentrated in vacuum. The residue was extracted with dichloromethane (50 mL × 3). The organic layer was separated and dried by anhydrous Na_2SO_4. After concentrated in vacuum, the residue was purified by column chromatography on silica gel (PE: EA = 10:1). A yellow solid **M9** 5.4 g was obtained in 42.2% yield. ^1H NMR (400 MHz, DMSO-d_6, ppm), δ: 11.35 (s, 1 H, -OH), 8.18 (d, J = 7.6 Hz, 1 H, Ar-H), 7.96 (d, J = 9.2 Hz, 1 H, Ar-H), 7.72 (d, J = 2.8 Hz, 1 H, Ar-H), 7.36 (s, 1 H, Ar-H), 7.29 (d, J = 8.0 Hz, 1 H, Ar-H), 7.15 (dd, J_1 =8.8 Hz, J_2 =2.8 Hz, 1 H, Ar-H), 3.86 (s, 3 H, -O-CH_3), 1.32 (s, 12 H, -CH_3). ^{13}C NMR (151 MHz, $CDCl_3$, ppm), δ:172.801, 156.129, 129.102, 125.008, 113.380, 112.400, 107.570, 55.876.

Synthesis and characterization of M10

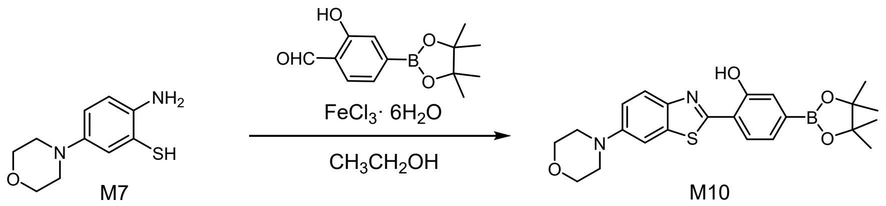

Scheme S8. Synthesis route of **M10**

To a 250 mL single necked bottle, **M7** (5.0 g, 23.8 mmol, 1 eq), 4-formyl-3-hydroxyphenylboronic acid pinacol ester (7.1 g, 28.6 mmol, 1.2 eq) and ethanol (100 mL) were added. The mixture was heated to 95 °C for 4 h. The organic solvents were removed by vacuum rotary evaporation, the precipitate was dissolved in THF solution, and was added dropwise into $FeCl_3 \cdot 6H_2O$ (23.8 g, 95.2 mmol, 4 eq) dissolved in ethanol, the mixture was heated at 80 °C for 2 h, then the organic layer was concentrated in vacuum. The residue was extracted with dichloromethane (50 mL × 3), The organic layer was separated and dried by anhydrous Na_2SO_4. After concentrated in vacuum, the residue was purified by column chromatography on silica gel (PE: EA = 10:1). A yellow solid **M10** 4.5 g was obtained in 42.1% yield. ^1H NMR (400 MHz, DMSO-d_6, ppm), δ: 11.40 (s, 1 H, -OH), 8.12 (d, J = 8.0 Hz, 1 H, Ar-H), 7.90 (d, J = 8.8 Hz, 1 H, Ar-H), 7.60 (s, 1 H, Ar-H), 7.34 (s, 1 H, Ar-H), 7.27 (d, J = 8.4 Hz, 2 H, Ar-H), 3.78 (t, J = 4.4 Hz, 4 H, morpholine-CH_2-CH_2), 3.22 (t, J = 4.4 Hz, 4 H, morpholine-CH_2-CH_2), 1.31 (s, 12 H, -CH_3). ^{13}C NMR (151 MHz, $CDCl_3$, ppm), δ: 166.085, 156.731, 149.832, 145.837, 134.706, 127.177, 125.270, 123.951, 122.498, 119.142, 116.909, 106.426, 84.095, 66.819, 49.703,

24.820.

Synthesis and characterization of DH

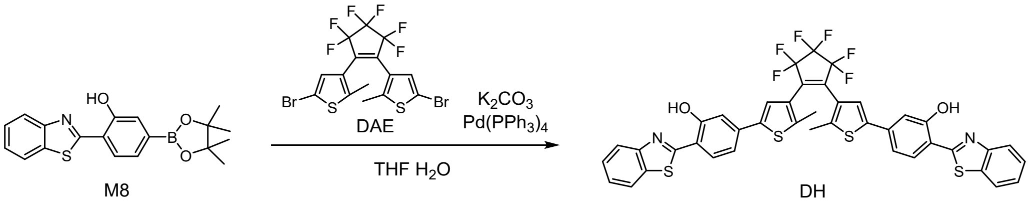

Scheme S9. Synthesis route of **DH**

To a 100 mL single necked bottle, DAE (1.0 g, 1.9 mmol, 1 eq), **M8** (1.7 g, 4.6 mmol, 2.4 eq), Pd $(PPh_3)_4$ (220.2 mg, 0.2 mmol, 0.1 eq), K_2CO_3 (790.1 mg, 5.7 mmol, 3 eq), THF (60 mL) and H_2O (20 mL) were added. The mixture was heated to 85 °C under nitrogen atmosphere for 8 h, then the organic layer was separated and concentrated in vacuum. The organic layer was separated and dried by anhydrous Na_2SO_4. After concentrated in vacuum, the crude product was purified by column chromatography (DCM: PE = 1:1) to afford the desired product **DH** 500 mg (yield: 32.3%). ^{1}H NMR (400 MHz, $CDCl_3$, ppm) δ: 12.56 (s, 1 H, -OH), 8.00 (d, J = 8.0 Hz, 1 H, Ar-H), 7.92 (d, J = 8.0 Hz, 1 H, Ar-H), 7.70 (d, J = 8.4 Hz, 1 H, Ar-H), 7.53 (t, J = 7.6 Hz, 1 H, Ar-H), 7.42 (t, J = 7.6 Hz, 1 H, Ar-H), 7.40 (s, 1 H, Ar-H), 7.29 (s, 1 H, thiophene-H), 7.16 (d, J = 8.4 Hz, 1 H, Ar-H), 2.04 (s, 3 H, -CH_3). ^{13}C NMR (151 MHz, $CDCl_3$, ppm), δ: 168.58, 158.29, 142.63, 141.09, 137.37, 137.34, 132.52, 129.07, 126.92, 126.05, 125.72, 123.75, 122.07, 121.58, 116.82, 116.15, 114.26, 14.72. High-resolution mass spectrometry (ESI positive ion mode for $[M+H]^+$): Calcd. for $C_{43}H_{27}N_2O_4F_6S_4$: 877.0775; Found: 877.0758.

Synthesis and characterization of DH-OMe

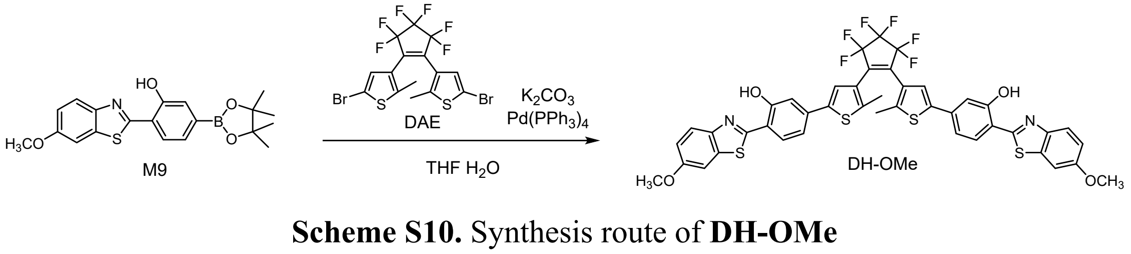

Scheme S10. Synthesis route of **DH-OMe**

To a 100 mL single necked bottle, DAE (1.0 g, 1.9 mmol, 1 eq), **M9** (1.7 g, 4.6 mmol, 2.4 eq), Pd $(PPh_3)_4$ (220.2 mg, 0.2 mmol, 0.1 eq), K_2CO_3 (790.1 mg, 5.7 mmol, 3 eq), THF (60 mL) and H_2O (20

mL) were added. The mixture was heated to 85 °C under nitrogen atmosphere for 8 h, then the organic layer was separated and concentrated in vacuum. The organic layer was separated and dried by anhydrous Na_2SO_4. After concentrated in vacuum, the crude product was purified by column chromatography (DCM: PE = 1:1) to afford the desired product **DH-OMe** 630.3 mg (yield: 37.7%). ^{1}H NMR (400 MHz, $CDCl_3$, ppm), δ: 12.53 (s, 1 H, -OH), 7.88 (d, J = 9.2 Hz, 1 H, Ar-H), 7.63 (d, J = 8.0 Hz, 1 H, Ar-H), 7.39 (s, 1 H, thiophene-H), 7.36 (d, J = 2.8 Hz, 1 H, Ar-H), 7.27 (d, J = 2.0 Hz, 1 H, Ar-H), 7.16-7.09 (m, 2 H, Ar-H), 3.91 (s, 3 H, -O-CH_3), 2.00 (s, 3 H, -CH_3). ^{13}C NMR (151 MHz, $CDCl_3$, ppm), δ: 169.83, 166.10, 158.16, 157.91, 146.33, 142.43, 141.22, 136.75, 128.69, 126.03, 123.53, 122.76, 116.77, 116.51, 116.03, 114.15, 104.24, 55.90, 53.56, 14.71. High-resolution mass spectrometry (ESI positive ion mode for $[M+H]^+$): Calcd. for $C_{43}H_{27}N_2O_4F_6S_4$: 877.0775; Found: 877.0758.

Synthesis and characterization of DH-Mor

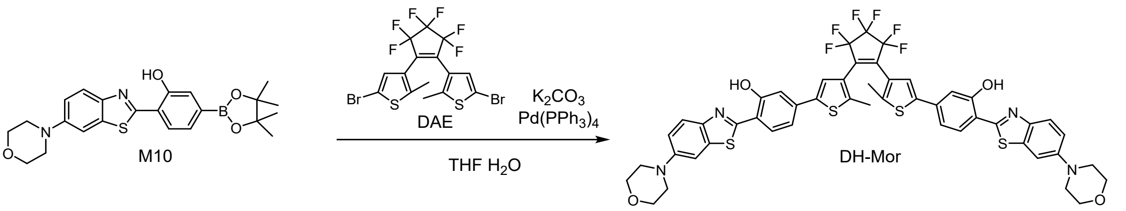

Scheme S11. Synthesis route of **DH-Mor**

To a 100 mL single necked bottle, DAE (1.0 g, 1.9 mmol, 1 eq), **M10** (1.9 g, 4.6mmol, 2.4eq), Pd $(PPh_3)_4$ (220.2 mg, 0.2 mmol, 0.1 eq), K_2CO_3 (790.3 mg, 5.7 mmol, 3 eq), THF (60 mL) and H_2O (20 mL) were added. The mixture was heated to 85 °C under nitrogen atmosphere for 8 h, then the organic layer was separated and concentrated in vacuum. The organic layer was separated and dried by anhydrous Na_2SO_4. After concentrated in vacuum, the crude product was purified by column chromatography (DCM: PE = 1:1) to afford the desired product **DH-Mor** 610.5 mg (yield: 32.6%). ^{1}H NMR (400 MHz, DMSO-d_6, ppm), δ: 11.78 (s, 1 H, -OH), 8.08 (d, J = 8.8 Hz, 1 H, Ar-H), 7.90 (d, J = 8.0 Hz, 1 H, Ar-H), 7.62 (s, 1 H, Ar-H), 7.59 (d, J = 2.4 Hz, 1 H, Ar-H), 7.35-7.21 (m, 3 H, Ar-H and thiophene-H), 3.77 (t, J = 4.8 Hz, 4 H, morpholine-CH_2-CH_2), 3.21 (t, J = 4.8 Hz, 4 H, morpholine-CH_2-CH_2), 2.02 (s, 3 H, -CH_3). ^{13}C NMR (101 MHz, $CDCl_3$, ppm), δ: 157.89, 146.05, 142.40, 141.25, 136.61, 134.46, 132.15, 128.64, 126.03, 123.51, 122.46, 122.40, 117.01, 116.58, 114.13, 114.01, 77.36, 77.04, 76.72, 66.77, 49.84, 14.73. High-resolution mass spectrometry (ESI positive ion mode

for [M+H]+): Calcd. for $C_{49}H_{39}N_4O_4F_6S_4$: 989.1784; Found: 989.1758.

Synthesis and characterization of DH-Mor-GSH

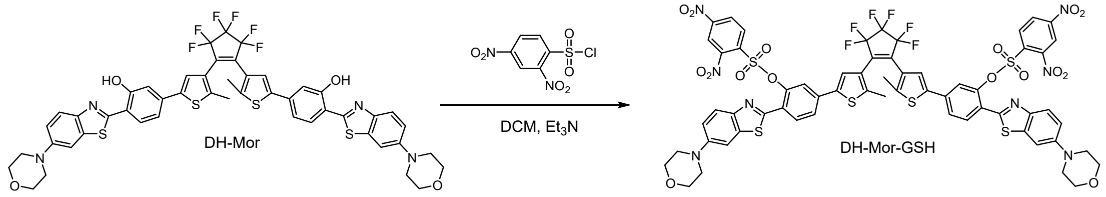

Scheme S12. Synthesis route of **DH-Mor-GSH**

To a 50 mL single necked bottle, **DH-Mor** (100.3 mg, 0.1 mmol, 1 eq) and dry dichloromethane were added. The mixture was cooled in ice water bath, and then triethylamine (51.2 mg, 0.5 mmol, 5 eq) was stirred and stirred for 5 min. 2,4-dinitrobenzenesulfonyl chloride (134.3 mg, 0.5 mmol, 5 eq) was dissolved in dry dichloromethane and added to the mixture. The reaction system was stirred at room temperature for 2 h. After the reaction, water was added, extracted with dichloromethane three times (50 mL × 3). The organic layer was separated and dried by anhydrous Na_2SO_4. After concentrated in vacuum, the crude product was separated by silica gel column chromatography (DCM: PE = 1:1) to give 41.3 mg yellow solid **DH-Mor-GSH** in 28.6% yield. ^{1}H NMR (400 MHz, $CDCl_3$, ppm), δ: 8.24 (d, J = 2.2 Hz, 1 H, Ar-H), 7.95 (d, J = 8.8 Hz, 1 H, Ar-H), 7.88 (d, J = 8.6 Hz, 1 H, Ar-H), 7.80 (d, J = 8.0 Hz, 1 H, Ar-H), 7.63 (d, J = 8.6 Hz, 1 H, Ar-H), 7.58 (d, J = 8.0 Hz, 1 H, Ar-H), 7.54 (s, 1 H, Ar-H), 7.33 (s, 1 H, thiophene-H), 7.12 (s, 1 H, Ar-H), 7.03 (d, J = 9.1 Hz, 1 H, Ar-H), 3.88 (s, 4 H, morpholine-CH_2-CH_2), 3.21 (s, 4 H, morpholine-CH_2-CH_2), 2.04 (s, 3 H, -CH_3). ^{13}C NMR (151 MHz, DMSO-d_6, ppm), δ: 161.77, 156.66, 149.64, 147.92, 146.80, 145.37, 142.67, 141.10, 137.57, 136.23, 135.69, 133.52, 132.23, 132.03, 129.37, 128.47, 125.59, 124.00, 122.61, 121.47, 118.57, 117.17, 116.75, 113.26, 106.38, 66.46, 49.18, 14.58. High-resolution mass spectrometry (ESI positive ion mode for [M+Na]+): Calcd. for $C_{61}H_{42}N_8O_{16}F_6S_6Na$: 1471.0839; Found: 1471.0902.

Synthesis and characterization of DH-Mor-*β*-Gal

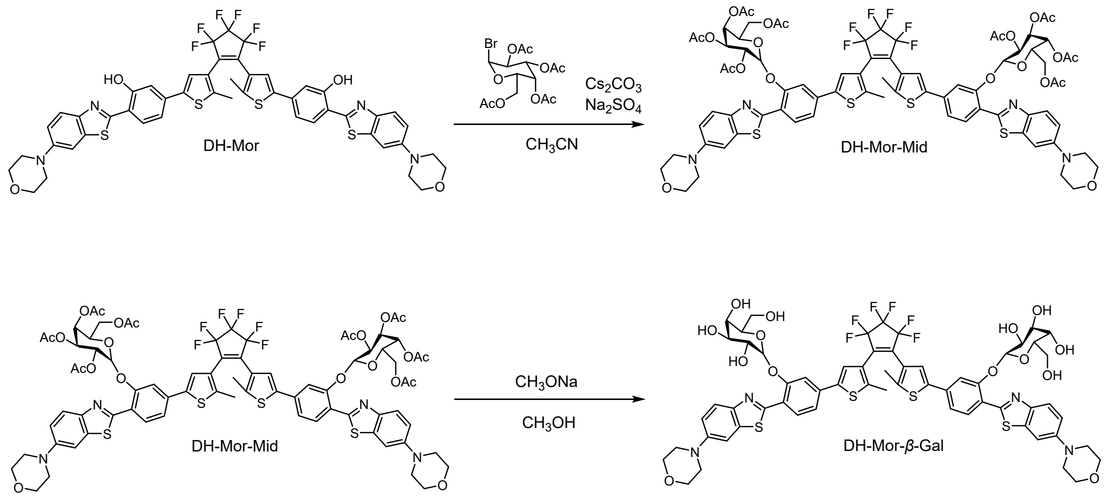

Scheme S13. Synthesis route of **DH-Mor-*β*-Gal**

To a 550 mL single necked bottle, **DH-Mor** (100.6 mg, 0.1 mmol, 1 eq), Na_2SO_4 (71.4 mg, 0.5 mmol, 5 eq), Cs_2CO_3 (164 mg, 0.5 mmol, 5 eq), CH_3CN (10 mL), and 2,3,4,6-Tetra-O-acetyl-alpha-D-galactopyranosyl bromide (207.8 mg, 0.5 mmol, 5 eq) were added. The mixture was stirred at room temperature under argon protection for 12 h. The precipitate was removed by filtration and the solvent was concentrated in vacuum to give the intermediate DH-Mor-Mid. Furthermore, compound **DH-Mor-Mid** (100.1 mg, 0.06 mmol, 1 eq) was dissolved in the solution of DCM 20% and CH_3OH 80% (20 mL) at 0 °C. Then sodium methylate (16.4 mg, 0.3 mmol, 5 eq) was added to the reaction. After refluxing for 30 min, a yellow precipitate was precipitated and the yellow solid **DH-Mor-*β*-Gal** 52.4 mg was obtained after filtration (39.2% yield over two steps). ^{1}H NMR (400 MHz, DMSO-d_6, ppm), δ: 8.39 (d, J = 8.4 Hz, 1 H, Ar-H), 7.89 (d, J = 8.8 Hz, 1 H, Ar-H), 7.66 (s, 1 H, thiophene-H), 7.53 (d, J = 8.4 Hz, 1 H, Ar-H), 7.46 (d, J = 8.4 Hz, 1 H, Ar-H), 7.25 (d, J = 8.0 Hz, 1 H, Ar-H), 5.29 (d, J = 7.6 Hz, 1 H, pyran-H), 5.16 (d, J = 5.6 Hz, 1 H, pyran-H), 4.99 (d, J = 5.8 Hz, 1 H, pyran-H), 4.72 (t, J = 5.6 Hz, 1 H, pyran-H), 4.66 (d, J = 4.0 Hz, 1 H, pyran-H), 3.93 (m, 1 H, -OH), 3.81-3.70 (m, 6H, morpholine-CH_2-CH_2, -CH_2-OH), 3.53 (m, 3 H, -OH), 3.21 (s, 4 H, morpholine-CH_2-CH_2), 2.03 (s, 3 H, -CH_3). ^{13}C NMR (101 MHz, $CDCl_3$, ppm), δ: 148.53, 146.88, 145.78, 142.49, 138.34, 136.66, 135.64, 133.11, 131.49, 130.86, 128.72, 125.25, 125.17, 124.94, 124.13, 123.56, 122.63, 120.44, 118.52, 115.68, 76.32, 76.21, 76.00, 75.68, 65.64, 48.24, 28.68, 13.83. High-resolution mass spectrometry (ESI positive ion mode for $[M+Na]^+$): Calcd. for $C_{61}H_{58}N_4O_{14}F_6S_4Na$: 11335.2629; Found: 1335.2623.

1.4 Cell lines

The Hela, OVCAR3 and HepG2 cells were all propagated in T-25 flasks cultured at 37 °C under a humidity 5% CO_2 atmosphere. DMEM medium (BIOAGRIO/ Shanghai Yuli Biotechnology Co, Ltd), which were supplemented with 10% fetal bovine serum (FBS, BIOAGRIO) and 1% penicillin-streptomycin (10,000 U mL^{-1} penicillin and 10 mg mL^{-1} streptomycin, BIOAGRIO).

1.5 Western blot

The Hela cells, OVCAR3 cells and HepG2 cells were lysed with cell lysis buffer (Beyotime Institute of Biotechnology, Shanghai, China) assisted with 0.5 mM phenylmethanesulfonyl fluoride. After centrifugation (12,000 rpm, 15 min, 4 °C). Sample lysates were separated by sodium dodecyl sulfate-polyacrylamide gel electrophoresis (SDSPAGE) and transferred to polyvinylidene difluoride (PVDF) membranes. Membranes were blocked for 2 h with 5% nonfat milk in Tris-buffered saline at room temperature and then incubated with anti-beta-galactosidase rabbit polyclonal antibody (1:1000, Cell Signaling Technology, 12713) at 4 °C overnight. The membranes were then incubated with secondary antibody for 1 h. Protein bands were determined using an enhanced chemiluminescence detection kit and photographed by a GE Amersham Imager 600 imaging system.

1.6 In vitro cytotoxicity assay

The cell cytotoxicity of DH-Mor-GSH and DH-Mor-*β*-Gal to Hela cells were measured by Cell Counting Kit-8 (CCK-8) assay. Cells were plated in 96-well plates in 100 μL volume of DMEM medium with 10% FBS, at a density of 5×10^3 cells/well and incubated with desired concentrations of DH-Mor-GSH and DH-Mor-*β*-Gal for 24 h. 10 μL CCK-8 solution was added into each well, and further cultured for 1 h. The absorbance was measured at 450 nm with a Tecan GENios Pro multifunction reader (Tecan Group Ltd., Maennedorf, Switzerland). Each concentration was measured in triplicate and used in three independent experiments. The relative cell viability was calculated by the equation: cell viability (%) = ($OD_{treated}$-OD_{blank} /$OD_{control}$-OD_{blank}) × 100%.

1.7 Confocal laser scanning microscopy

The Hela, OVCAR3 and HepG2 cells at 1×10^5 cells/well were seeded onto glass-bottom petri dishes with complete medium (1.5 mL) for 6 h. Then the cells were exposed to desired concentration

10 μM of DH-Mor-GSH for 30 min, 20 μM of DH-Mor-*β*-Gal for 1h. PBS (pH 7.4) was used to wash cells for three times to clean the background. The images were then photographed by Leica TCS SP8 (63 × oil lens) with 405 nm as the excitation wavelength and 500-600 nm as the emission wavelength. To test the photoresponse performance of the probe within cells, cells were irradiated with 450 and 561 nm laser cycles in a dark room and photographed under a microscope, and the irradiation was repeated for at least three cycles.

1.8 STORM imaging protocol

Super-resolution STORM imaging was performed on a Nikon N-STORM microscope equipped with an Ti2-E inverted microscope, an Apochromat TIRF 100 × oil immersion lens with a numerical aperture of 1.49 (Nikon), an electron-multiplying charge-coupled device (EMCCD) camera (iXon3 DU-897E, Andor Technology), a quad band filter composed of a quad line beam splitter (zt405/488/561/640rpc TIRF, Chroma Technology Corporation), and a quad line emission filter (bright line HC 446, 523, 600, 677, Semrock, Inc.). Lasers used in experimental procedures: 488 Arlaser (50 mW: Melles Griot). The focus was kept stable during acquisition using a Nikon focus system. During the imaging experiment, the fluorescence state was reversibly turned on and off under visible light 488nm continuous to collect cell images, after acquiring 5000 frames (30 ms per frame), the Gauss algorithm was used to reconstruct the images. To image intracellular GSH or *β*-Gal, cells (on a glass bottom cell culture plate) were stained with DH-Mor-GSH (10 μM) for 30 min (DH-Mor-*β*-Gal (20 μM) for 60 min), followed by three washes with PBS buffer, finally, the cells were fixed with 4% PFA. Super-resolution images were reconstructed using the N-STORM analysis module of NIS-Elements Ar.

1.9 Data analysis

We used Image J to calculate average fluorescence intensity of different cells. For localization number density, we reconstructed the raw image data using Nikon N-STORM Analysis Software. The area of the cell was measured by setting the scale in Image J. Then the localization density could be calculated by dividing the total localization number by the cell area. Analyzed cells were obtained from three replicates. Statistical significances and sample sizes in all graphs are indicated in the corresponding figure legends.

1.10 The preparation of PSt-b-PEO block copolymer micelles

The amphiphilic block copolymer PSt_{38k}-b-PEO_{11k} and DHs were initially dissolved in chloroform at a concentration of 10 mg/mL and 1.0 mg/mL, respectively. After complete dissolution, 0.1 mL solution of block copolymer PSt_{38k}-b-PEO_{11k} and 0.02 mL solution of **DHs** were mixed with 1 mL of SDS aqueous solution (1.0 mg/mL). After assuring a homogeneous mixture, chloroform was evaporated slowly in the atmosphere in 24 h. The residue solution was centrifuged at 5000 rpm for 10 minutes and the precipitate was dispersed in water affording cylindrical micelles.[1]

1.11 The fluorescence quantum yield measurement

The relative fluorescence quantum yields (Φ_u) were determined by a standard method[2-3], which involves measuring the sample's emission efficiency against a reference compound with a known quantum yield. The relative quantum yield was calculated using the following formulas. The excitation wavelength for fluorescence is 405 nm.

$$\Phi_u = \Phi_s \times (F_u/F_s) \times (A_s/A_u).$$

Φ: fluorescent quantum yield; F: fluorescence integral region; A: absorbance; u: sample; s: reference compound. Reference Compound: Rhodamine 6G (Φ_s = 95% in water).

Procedure: a) Measure Absorbance: use a UV-Vis spectrophotometer to measure A_s and A_u at λ_{ex}. b) Record Fluorescence Emission Spectra: Collect emission spectra for both sample and reference under identical conditions (slit widths, detector gain, scan speed). c) Fluorescence Integral Region: Integrate the area under the emission curve (F_u and F_s). d) Calculate the quantum yield according to formula.

1.12 The photoisomerization quantum yield measurement

Photochromic quantum yields were measured according to the standard procedure reported in previous literatures[4-5]. The experimental steps are as follows:

For the photocyclization quantum yields (irradiation wavelength: 365 nm and 450 nm) of DHs, potassium ferrioxalate ($K_3[Fe(C_2O_4)_3]$) was used as reference compound. 2 mL DMSO solution of open form DHs (1×10^{-4} M, 2×10^{-4} M, 3×10^{-4} M) was irradiated with the same light source, and the absorption changes at detection wavelength was measured immediately. Time-resolved absorption spectra were recorded using a Maya2000 spectrometer with SpectraSuite software, with a measurement interval of 0.1 s. The photocyclization quantum yields can be calculated via formula 1.

$$\Phi_{o-c} = \frac{\frac{dA}{dt} * V}{\left(1 - 10^{-lc\varepsilon_{o-i}}\right) * \varepsilon_{c-d} * I_0} \quad 1$$

dA/dt is the change rate of absorbance upon irradiation at excitation wavelength (Figure S4-S6), which was obtained by linearly fitting the time-resolved absorption diagram at the early stage of the reaction (5% of conversion rate) to avoid the interference of photoproducts/inner filter effects; *V* is the volume of liquid to be measured; *l* is the optical range; *c* is the initial concentration of the open form of DHs; $\varepsilon_{o\text{-}i}$ is the molar extinction coefficients of the open form at the wavelengths of the excitation light; $\varepsilon_{c\text{-}d}$ is the molar extinction coefficients at the detection wavelength (the detection wavelength at the λ_{max} of the closed form) of the closed form; I_0 is the light intensity (the error of direct measurement of I_0 is generally high, thus the reference compound $K_3[Fe(C_2O_4)_3]$ can be introduced for parallel experiments to invert the I_0).

Reference compound $K_3[Fe(C_2O_4)_3]$: i) 2 mL of 0.006 M (for 365 nm) or 0.012 M (for 450 nm) $K_3[Fe(C_2O_4)_3]$ solution in 0.05 M H_2SO_4 was irradiated for 180 s; ii) 0.5 mL phenanthroline (0.1 wt % in 0.05 M H_2SO_4 / 1.6M NaOAc) were added; iii) For $K_3[Fe(C_2O_4)_3]$, the detection wavelength is set at 510 nm, ε_{510nm} is 11100 M^{-1} cm^{-1}, Φ_{irr} is the quantum yield at the irradiation wavelength (1.21 for 365 nm and 1.07 for 450 nm).

For the photocycloreversion quantum yields (irradiation wavelength: 600 nm) of DHs, aberchrome 670 was used as reference compound. The test method is similar to the calculation of photocyclization quantum yields. The photocycloreversion quantum yields can be calculated via formula 2.

$$\Phi_{c-o} = \frac{\frac{dA}{dt} * V}{\left(1 - 10^{-lc\varepsilon_{c-i}}\right) * \varepsilon_{c-d} * I_0} \quad 2$$

dA/dt is the change rate of absorbance upon irradiating at excitation wavelength; *V* is the volume of liquid to be measured,;*l* is the optical range, *c* is the initial concentration of the closed form of DHs, $\varepsilon_{c\text{-}i}$ is the molar extinction coefficients of the closed form at the wavelengths of the excitation light; $\varepsilon_{c\text{-}d}$ is the molar extinction coefficients at the detection wavelength (the detection wavelength at the λmax of the closed form) of closed form; I_0 is the light intensity (the error of direct measurement of I_0 is

generally high, thus the reference compound aberchrome 670 can be introduced for parallel experiments to invert the I_0).

Reference compound aberchrome 670: i) 2 mL aberchrome 670 solution (10^{-4} M in toluene) was irradiated with 365nm light; ii) irradiated it back with 600 nm light, iii) measuring the absorbance at 519 nm before and after the 600 nm light irradiation. For aberchrome 670, the detection wavelength is set at 519 nm, $\varepsilon_{519\ nm}$ is 7760 M^{-1} cm^{-1}, Φ_{irr} is the quantum yield at the irradiation wavelength (0.15 for 600 nm).

Three independent dilutions were made with a concentration of 1×10^{-4} M, 2×10^{-4} M, 3×10^{-4} M in DMSO. As show in Figure S4-S6, we calculate the *dA/dt* (*dA/dt* can be obtained by linearly fitting the A-t diagram at the early stage of the reaction) for three samples with different concentrations, and the corresponding photochromic quantum yields are calculated by the formula (Table S1-S3), and the final values presented in Table 1 are the average of three measurements.

1.13 The fatigue resistance measurement

DH, **DH-OMe**, **DH-Mor** were diluted in DMSO to the concentration of 10 μM, respectively, then exposed to the light (Hamamatsu, LC8 Lightningcure, 200 W) with magnetic stirring the solutions. The absorption spectrum was recorded at the beginning and regular time intervals. The photostability experiment was repeated ten times independently.[6]

2. Supporting Figures

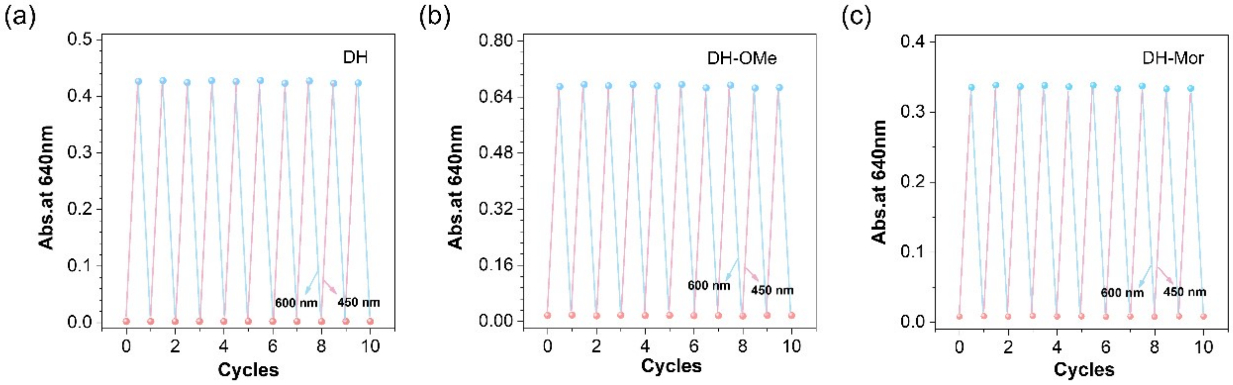

Figure S1. (a) Absorption changes of **DH**, **DH-OMe**, and **DH-Mor** at 640 nm upon alternate irradiation with 450 nm and 600 nm for 10 cycles.

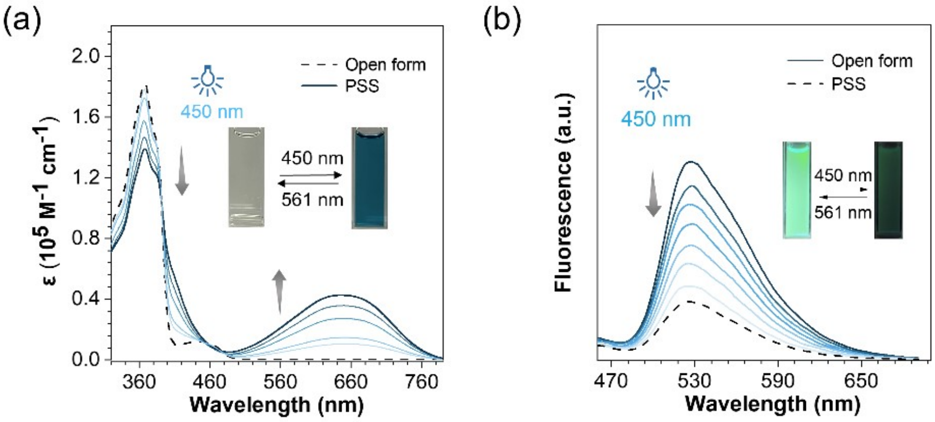

Figure S2. Absorption (a) and fluorescence (b) spectra of DH (λ_{ex} = 390 nm) under irradiation of visible light (λ = 450 nm) in DMSO solution. Inset images show the color changes.

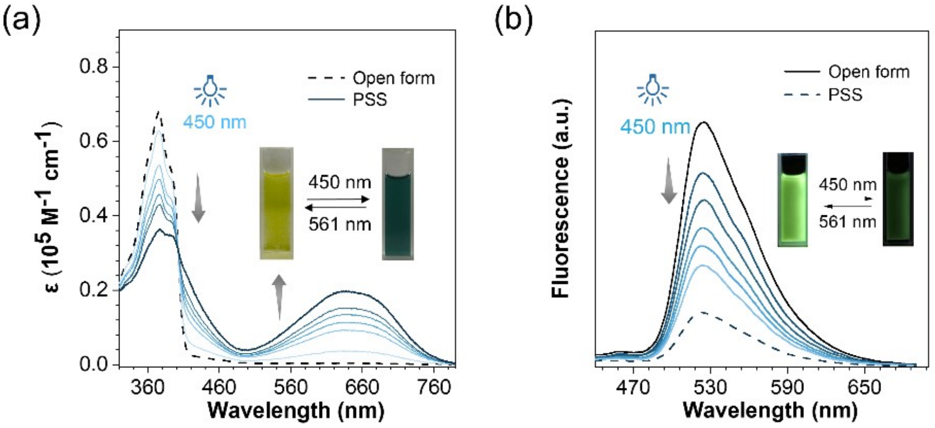

Figure S3. Absorption (a) and fluorescence (b) spectra of DH-OMe (λ_{ex} = 390 nm) under irradiation of visible light (λ = 450 nm) in DMSO solution. Inset images show the color changes.

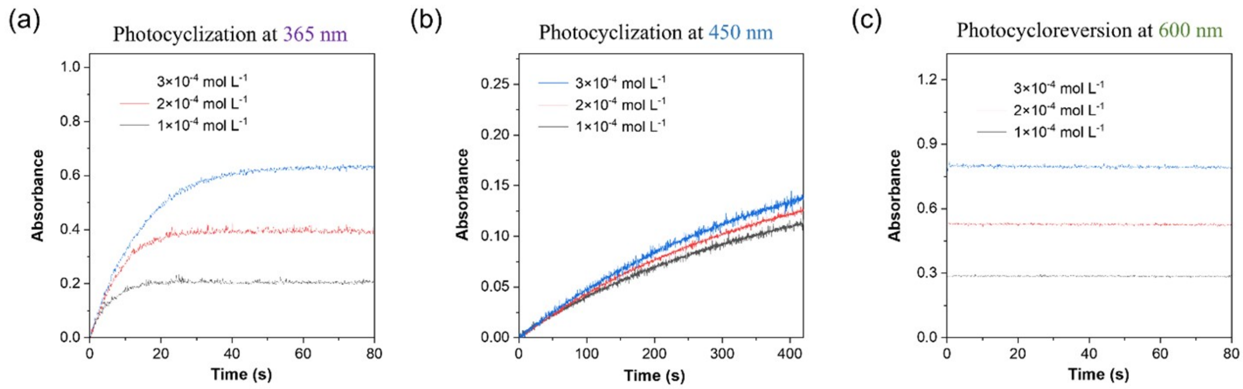

Figure S4. Absorption changes of **DH** in DMSO solution upon irradiation at different wavelengths. (a) Irradiated at 365 nm. (b) Irradiated at 450 nm. (c) Irradiated at 600 nm. The absorption was monitored at 640 nm for all experiments (a-c).

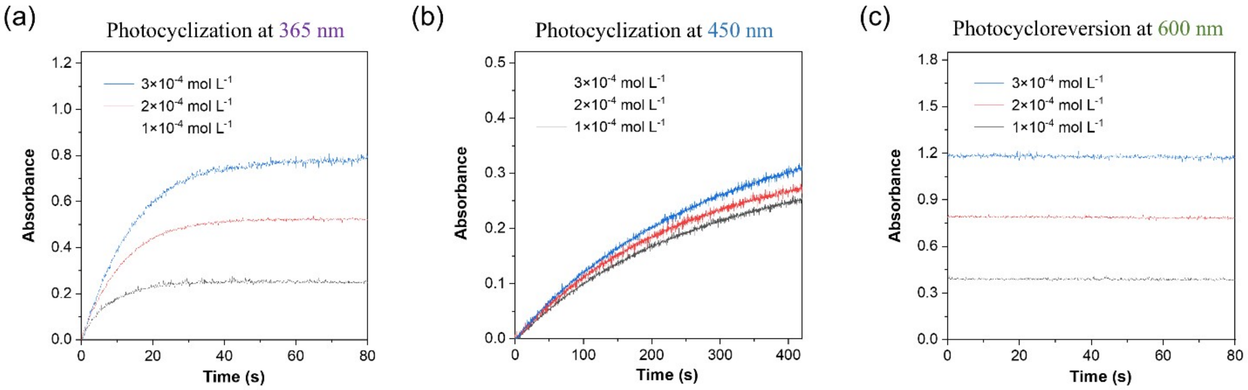

Figure S5. Absorption changes of **DH-OMe** in DMSO solution upon irradiation at different wavelengths. (a) Irradiated at 365 nm. (b) Irradiated at 450 nm. (c) Irradiated at 600 nm. The absorption was monitored at 640 nm for all experiments (a-c).

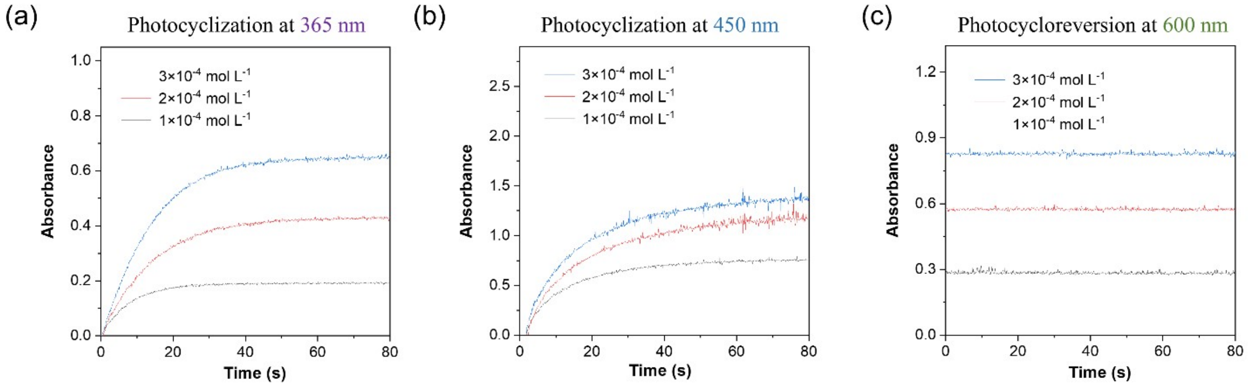

Figure S6. Absorption changes of **DH-Mor** in DMSO solution upon irradiation at different wavelengths. (a) Irradiated at 365 nm. (b) Irradiated at 450 nm. (c) Irradiated at 600 nm. The absorption was monitored at 640 nm for all experiments (a-c).

Table S1. Photocyclization and photocycloreversion quantum yields data of DH

Compound	Photocyclization				Photocycloreversion			
	λ_{irr}[b] [nm]	c [mol L^{-1}]	$\Phi_{o\text{-}c}$[c] [%]	$\Phi_{o\text{-}c}$[c] [%]	λ_{irr}[b] [nm]	c [mol L^{-1}]	$\Phi_{c\text{-}o}$[d] [%]	$\Phi_{c\text{-}o}$[d] [%]
DH	365	1×10^{-4}	35	37	600	1×10^{-4}	7.1	7.6
		2×10^{-4}	37			2×10^{-4}	7.7	
		3×10^{-4}	39			3×10^{-4}	8.1	
	450	1×10^{-4}	25	27				
		2×10^{-4}	27					
		3×10^{-4}	28					

Table S2. Photocyclization and photocycloreversion quantum yields data of DH-OMe

Compound	Photocyclization				Photocycloreversion			
	λ_{irr}[b] [nm]	c [mol L^{-1}]	$\Phi_{o\text{-}c}$[c] [%]	$\Phi_{o\text{-}c}$[c] [%]	λ_{irr}[b] [nm]	c [mol L^{-1}]	$\Phi_{c\text{-}o}$[d] [%]	$\Phi_{c\text{-}o}$[d] [%]
DH-OMe	365	1×10^{-4}	35	40	600	1×10^{-4}	5.3	5.5
		2×10^{-4}	40			2×10^{-4}	5.2	
		3×10^{-4}	44			3×10^{-4}	5.9	
	450	1×10^{-4}	26	29				
		2×10^{-4}	29					
		3×10^{-4}	31					

Table S3. Photocyclization and photocycloreversion quantum yields data of DH-Mor

Compound	Photocyclization				Photocycloreversion			
	λ_{irr}[b] [nm]	c [mol L^{-1}]	$\Phi_{o\text{-}c}$[c] [%]	$\Phi_{o\text{-}c}$[c] [%]	λ_{irr}[b] [nm]	c [mol L^{-1}]	$\Phi_{c\text{-}o}$[d] [%]	$\Phi_{c\text{-}o}$[d] [%]
DH-Mor	365	1×10^{-4}	43	42	600	1×10^{-4}	2.1	2.3
		2×10^{-4}	38			2×10^{-4}	2.3	
		3×10^{-4}	44			3×10^{-4}	2.6	
	450	1×10^{-4}	34	36				
		2×10^{-4}	36					
		3×10^{-4}	39					

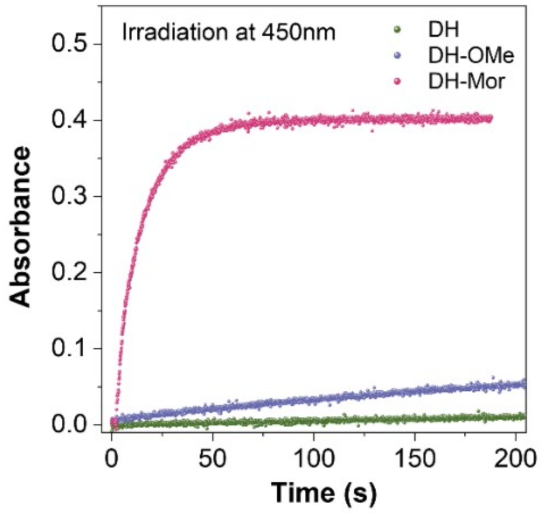

Figure S7. Absorbance variation curves of open form of **DH**, **DH-OMe**, and **DH-Mor** over the irradiation time of 450 nm visible light in DMSO solution.

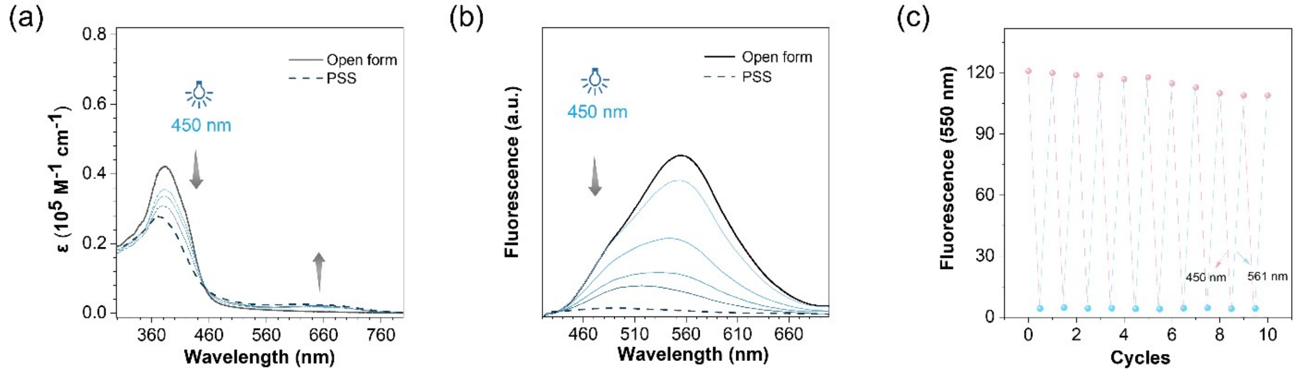

Figure S8. Absorption (a) and fluorescence (b) spectra of DH-Mor (λ_{ex} = 390 nm) under irradiation of visible light (λ = 450 nm) in aqueous PBS buffer solution (co-solubilized with 1% DMSO). (c) Fluorescence changes of DH-Mor in aqueous PBS buffer solution (co-solubilized with 1% DMSO) at 550 nm upon alternate irradiation with 450 nm and 561 nm for 10 cycles.

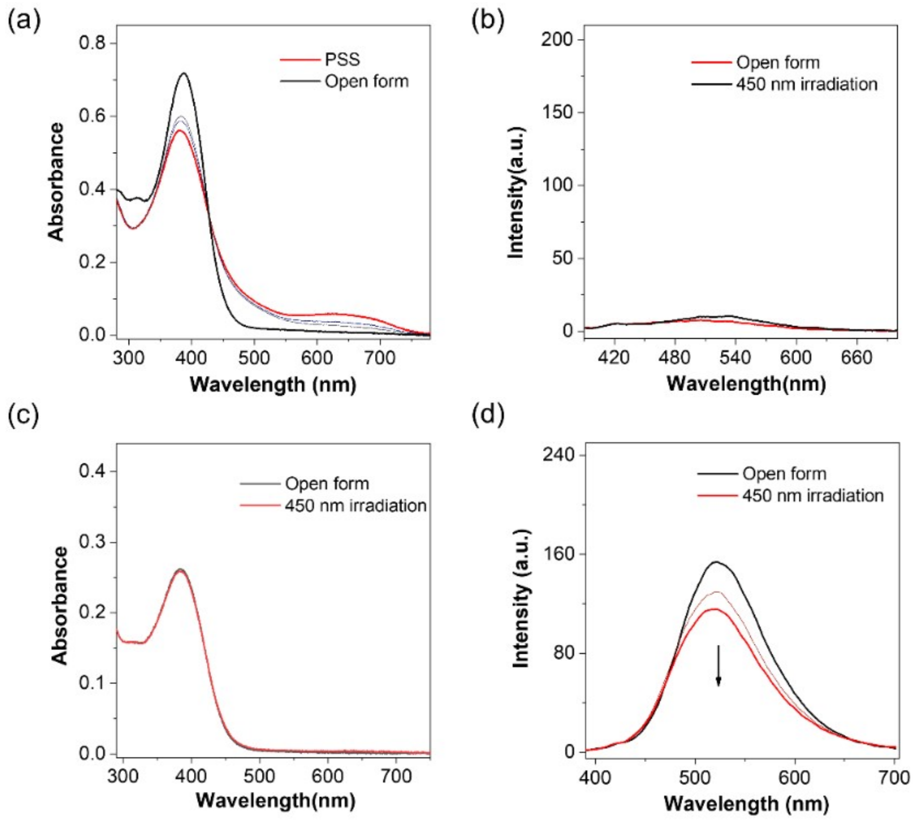

Figure S9. (a) Absorption and (b) fluorescence spectra of **DH-Mor-GSH** (10 μM) in PBS/DMSO solution (1: 9, v/v) upon 450 nm irradiation. (c) Absorption and (d) fluorescence spectra of **DH-Mor-GSH** (10 μM) in PBS/DMSO solution (1: 9, v/v) after adding GSH upon 450 nm irradiation.

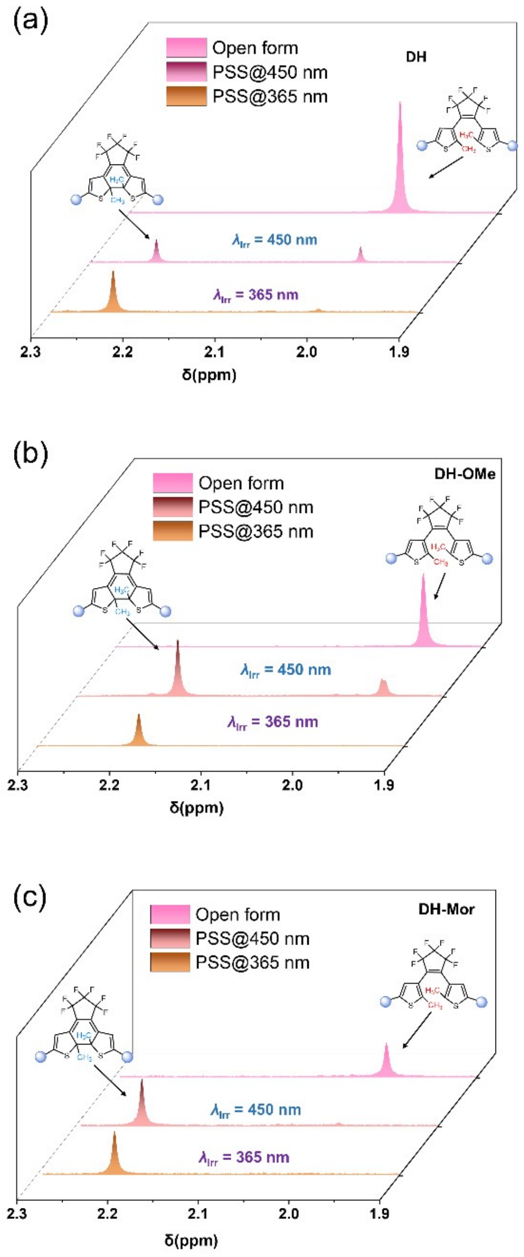

Figure S10. The photocyclization reaction yields of DHs estimated by comparing 1HNMR before and after 365 nm or 450 nm irradiation. The chemical shift of the methyl groups on the thiophene rings in o-DH-Mor and c-DH-Mor are 1.99 ppm and 2.22 ppm (o-DH: 2.00 ppm, c-DH: 2.22 ppm, o-DH-OMe: 1.96 ppm, c-DH-OMe: 2.18 ppm). By comparing their integration area values, the photocyclization reaction yield of DH-Mor upon 450 nm laser irradiation was calculated to be at least 93% (>99% upon 365 nm laser irradiation). the photocyclization reaction yield of DH upon 450 nm laser irradiation was calculated to be at least 62% (>93% upon 365 nm laser irradiation). The photocyclization reaction yield of DH-OMe upon 450 nm laser irradiation was calculated to be at least 69% (98% upon 365 nm laser irradiation).

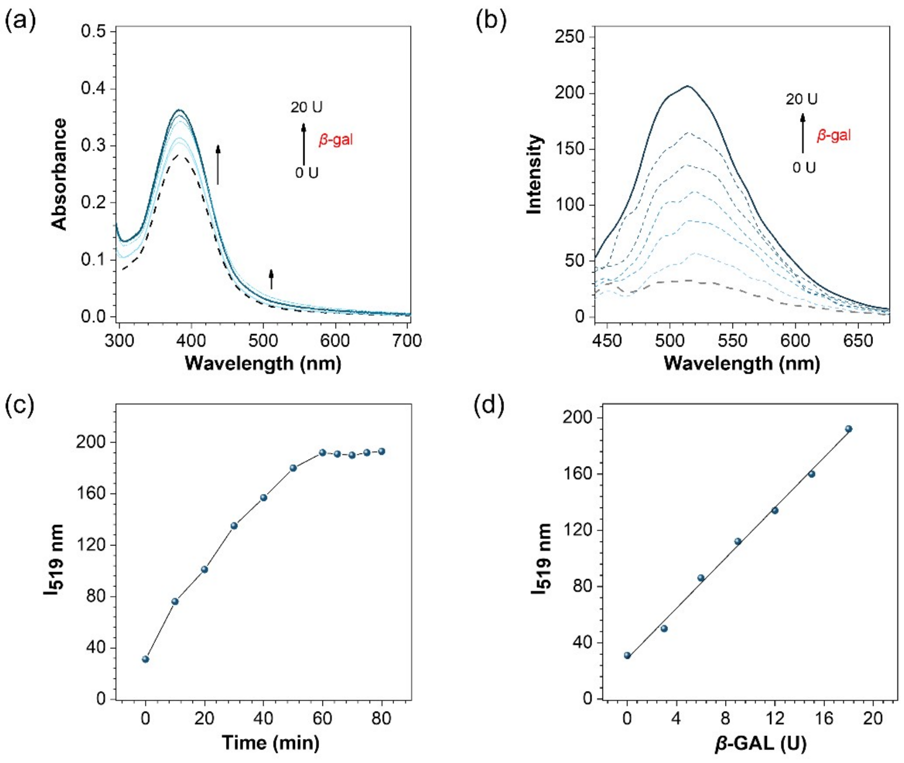

Figure S11. (a) Absorbance and (b) fluorescence spectra of **DH-Mor-*β*-Gal** (20 μM) incubation with *β*-Gal in a mixture solution of PBS/DMSO solution (8: 2, v/v) at 37 °C. (c) Time-dependent of $I_{519\ nm}$ (0−80 min) for **DH-Mor-*β*-Gal** incubation with *β*-Gal, (d) Emission changes plots of $I_{519\ nm}$ for **DH-Mor-*β*-Gal** as a function of *β*-Gal concentration (0-20 U).

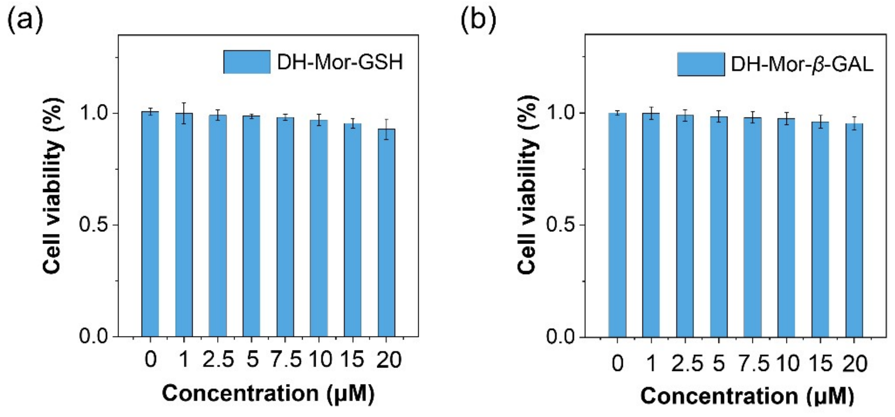

Figure S12. The cell viability of **DH-Mor-GSH** (a) and **DH-Mor-*β*-Gal** (b) at different concentrations. Data are shown as mean ± s.d. with n = 6. The CCK-8 experiment demonstrated low cytotoxicity with 92% cell viability after 24 hours of incubation at a concentration of 20 μM.

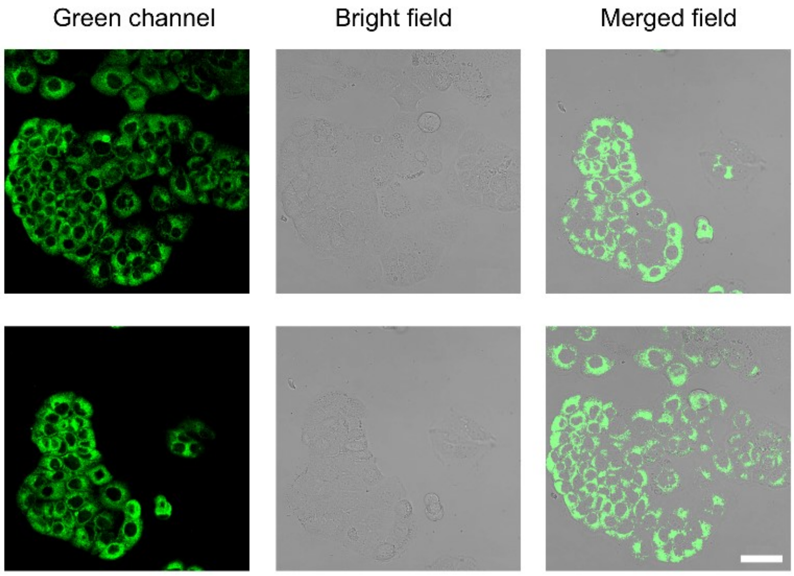

Figure S13. Confocal imaging of OVCAR3 cells indubated with **DH-Mor-*β*-Gal** (20 µM) for 60 min. Scale bar: 50 µm

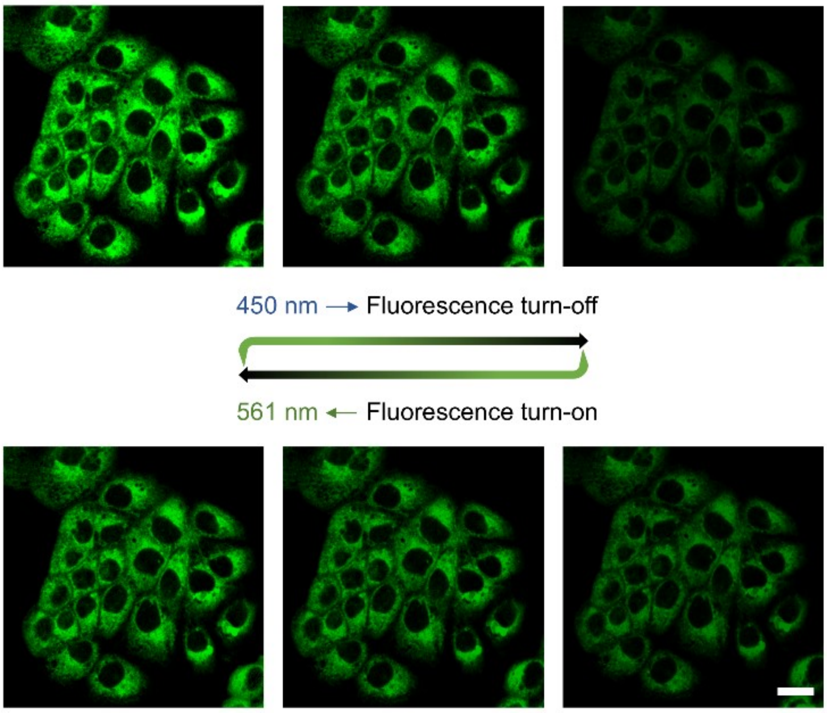

Figure S14. Photoactivation and photobleaching behaviors in fixed OVCAR3 cells recorded by confocal laser scanning microscopy. Scale bar: 20 µm

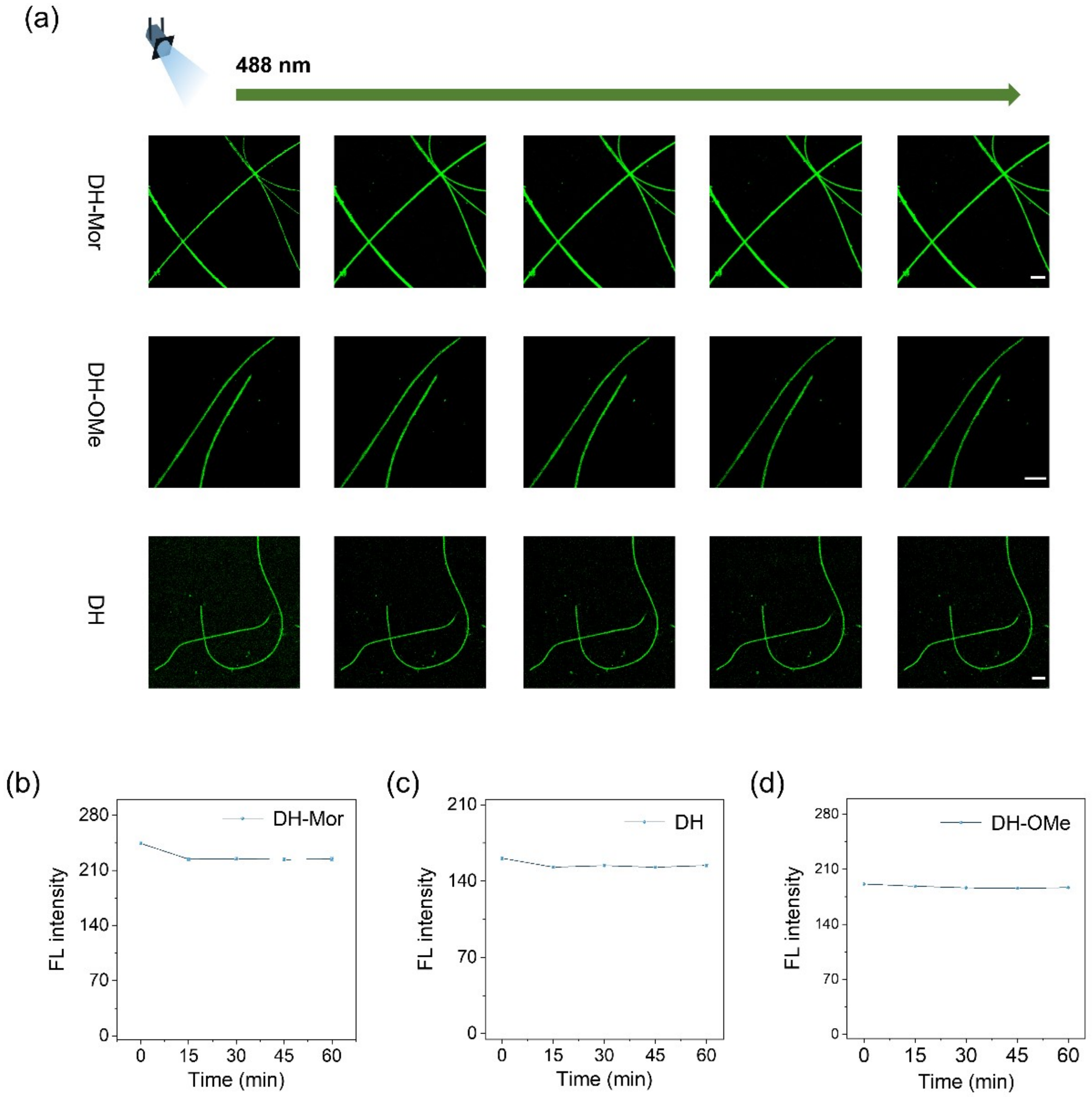

Figure S15. (a) Confocal imaging of **DH**, **DH-OMe**, **DH-Mor** in block polymer PSt_{38k}-b-PEO_{11k}. Scale bar: 10 μm. (b) Fluorescence intensity of block polymer of **DH-Mor** (b), **DH-OMe** (c), **DH** (d), under 488 nm laser irradiation. Data are shown as mean ± s.d. with n = 3.

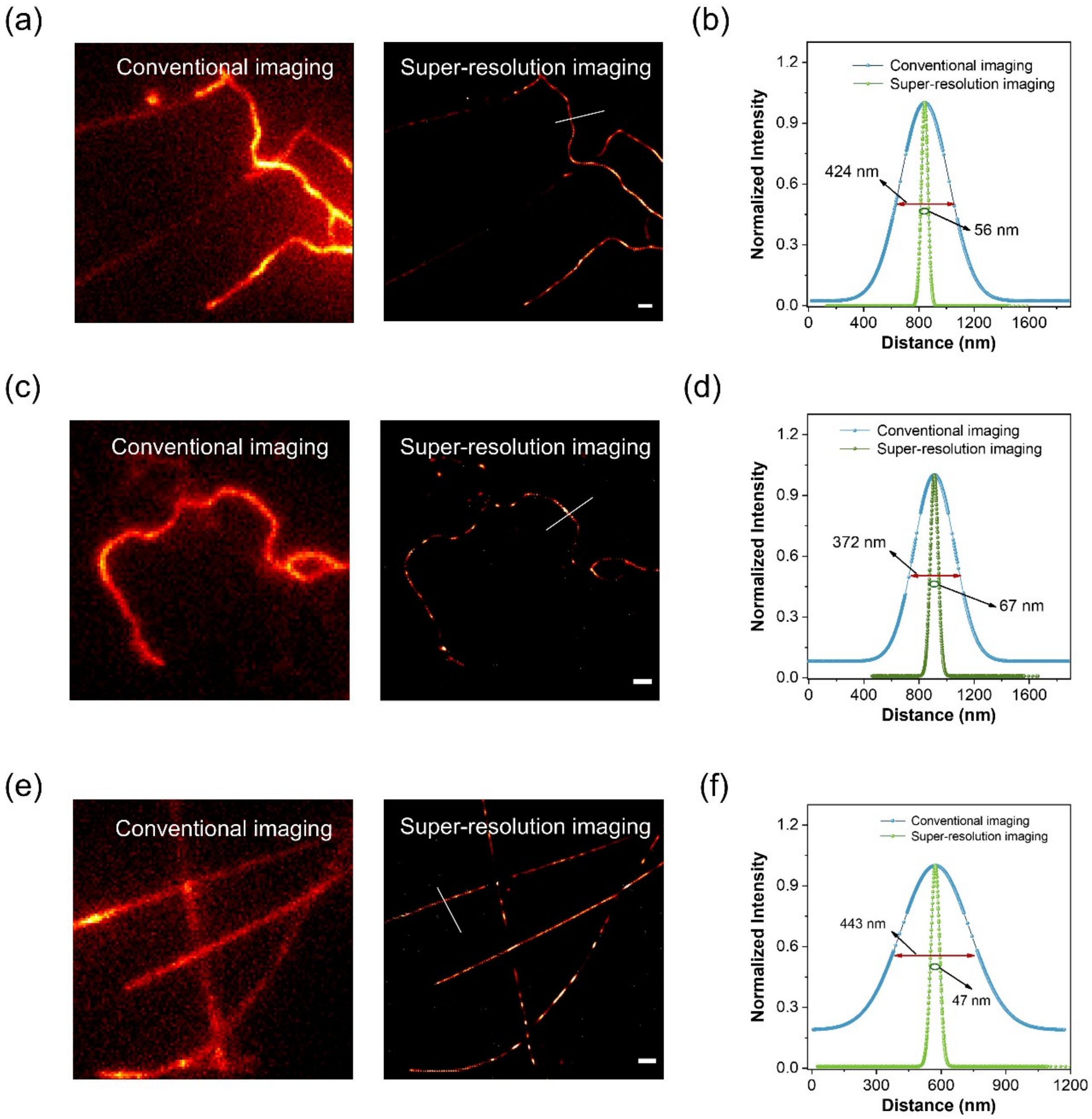

Figure S16. Conventional fluorescence image and super resolution imaging of PSt_{38k}-b-PEO_{11k} block polymer micelles labeled with **DH** (a), **DH-OMe** (c), **DH-Mor** (e). Scale bars, 5 μm. The cross-sectional profiles of conventional fluorescence and super resolution image of **DH** (b), **DH-OMe** (d), **DH-Mor** (f).

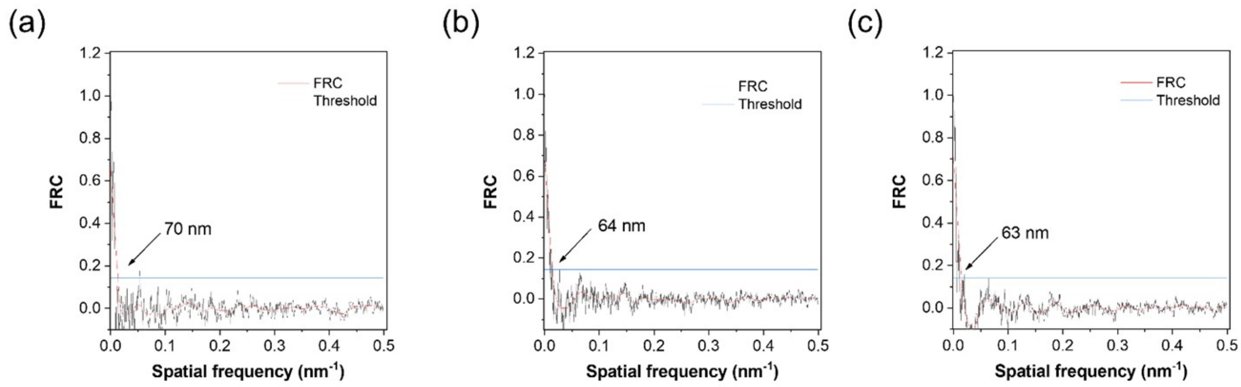

Figure S17. Fourier ring correlation (FRC) curve of the localizations presented in Figure S15 of super resolution imaging: (red line) a smoothed FRC curve and (blue line) a resolution threshold criterion 1/7 (≈ 0.143). The spatial resolution of the super-resolved image is calculated from the intersection between the FRC and the threshold, resulting in a value of about 70 nm of Figure S15 a, 64 nm of Figure S15 c, 63 nm of Figure S15 e.

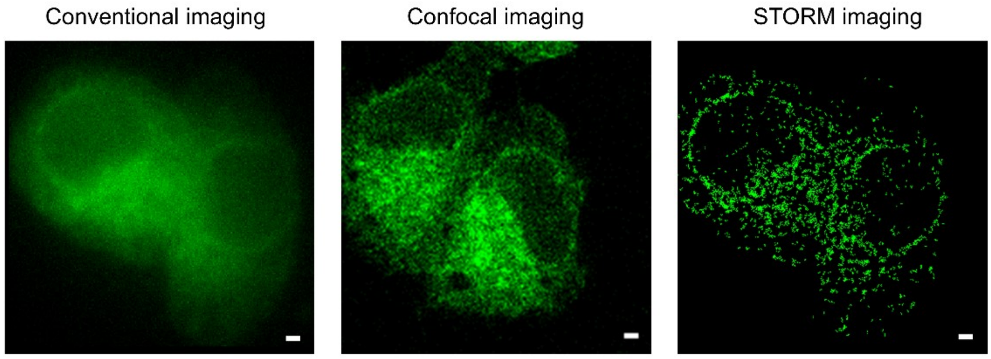

Figure S18. Conventional wide-field, confocal and STORM imaging of the Hela cells incubated with **DH-Mor-GSH** (10 μM) for 30 min. Scale bars, 2 μm.

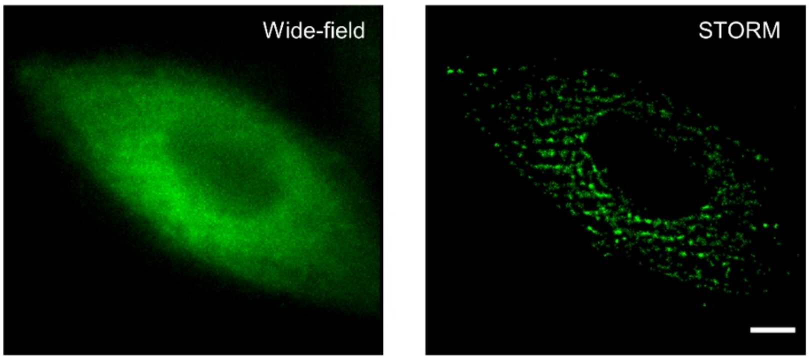

Figure S19. (a) Wide-field and (b) STORM images of the OVCAR3 cell incubated with **DH-Mor-*β*-Gal** (20 μM) for 60 min. Scale bars, 5 μm.

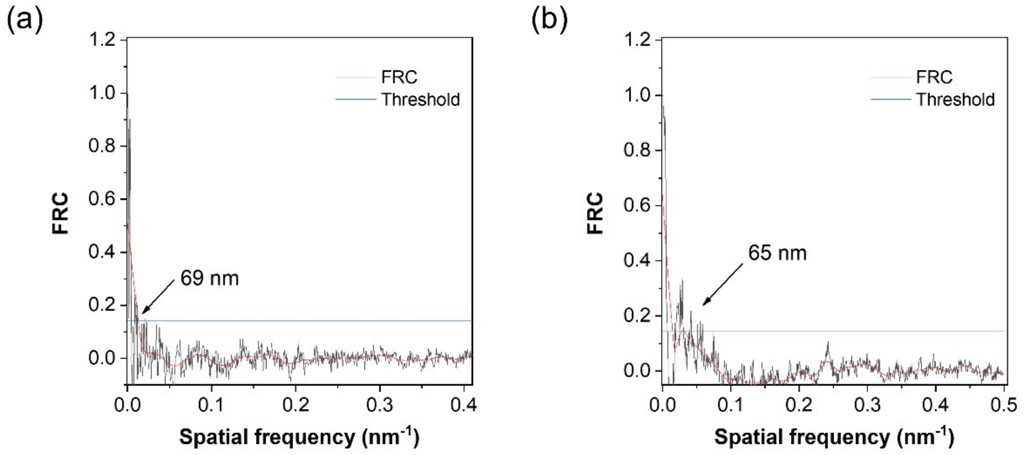

Figure S20. (a) Fourier ring correlation (FRC) curve of the localizations presented in Figure 4b and (b) Figure S18 of super resolution imaging: (red line) a smoothed FRC curve and (blue line) a resolution threshold criterion 1/7 (≈ 0.143). The spatial resolution of the super-resolved image is calculated from the intersection between the FRC and the threshold, resulting in a value of about 69 nm of Figure 4b, 65 nm of Figure S18.

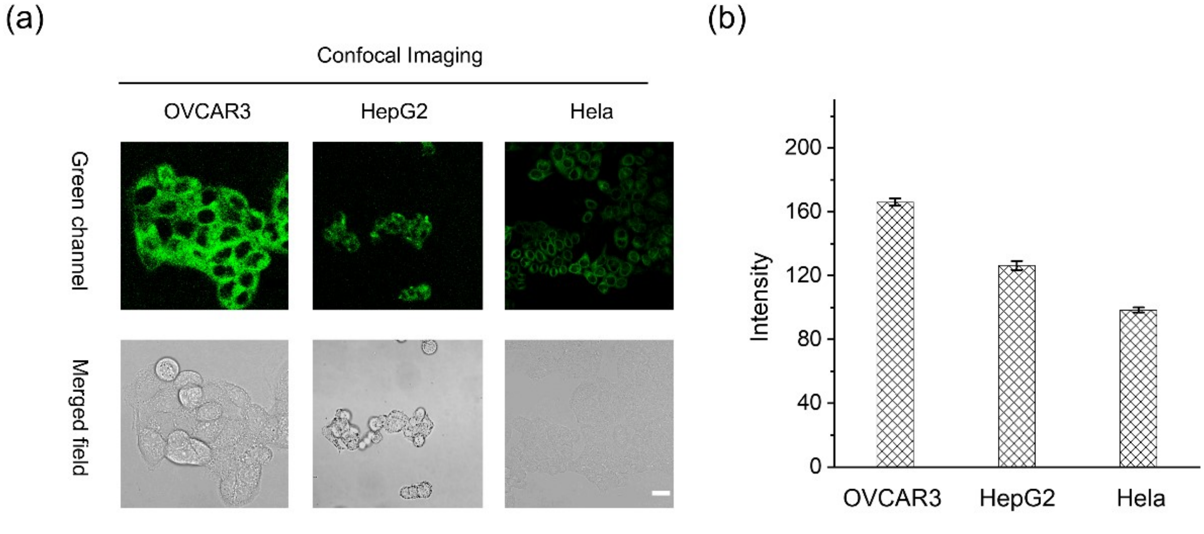

Figure S21. (a) Confocal images of endogenous *β*-Gal level in Hela, HepG2, OVCAR3 cells determined by DH-Mor-*β*-Gal (20 μM) staining for 60 min. Scale bars, 10 μm. (b) Quantified fluorescence intensities of cells as represented in confocal images of endogenous *β*-Gal level in Hela, HepG2, OVCAR3 cells.

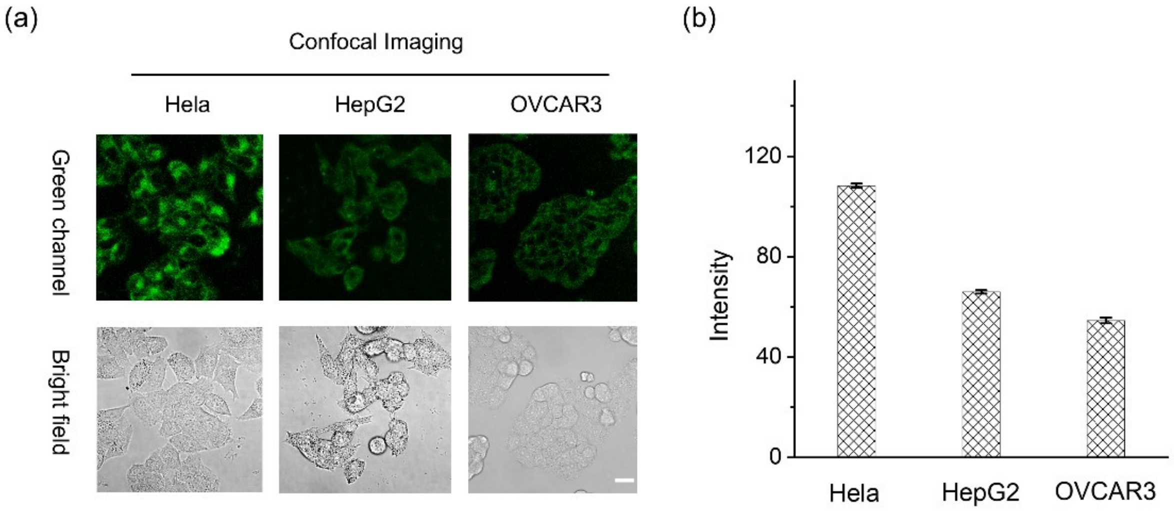

Figure S22. (a) Confocal images of endogenous GSH level in OVCAR3, HepG2, Hela cells determined by DH-Mor-GSH (10 μM) staining for 30 min. Scale bars, 10 μm. (b) Quantified fluorescence intensities of cells as represented in confocal images of endogenous GSH level in OVCAR3, HepG2 and Hela cells.

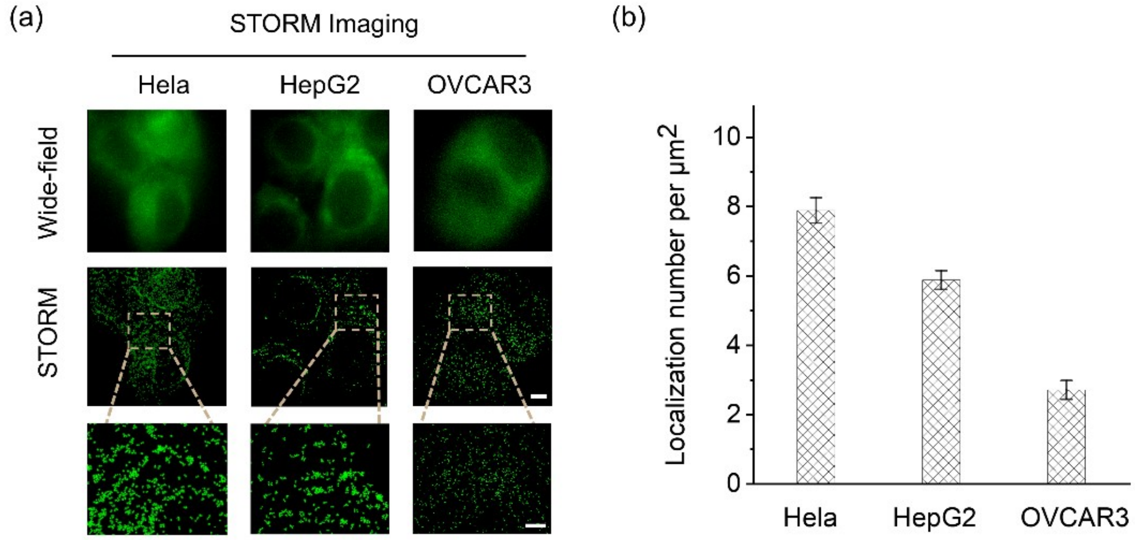

Figure S23. (a) STORM images of endogenous GSH level in Hela, HepG2 and OVCAR3 cells determined by **DH-Mor-GSH** (10 μM) staining for 30 min. Scale bars, 4 μm. Scale of enlarged area, 2 μm. (b) The number of GSH localizations per μm^2 on cells as represented in panel (a). Error bars represent standard deviation, n= 3.

3. Characterization of compounds

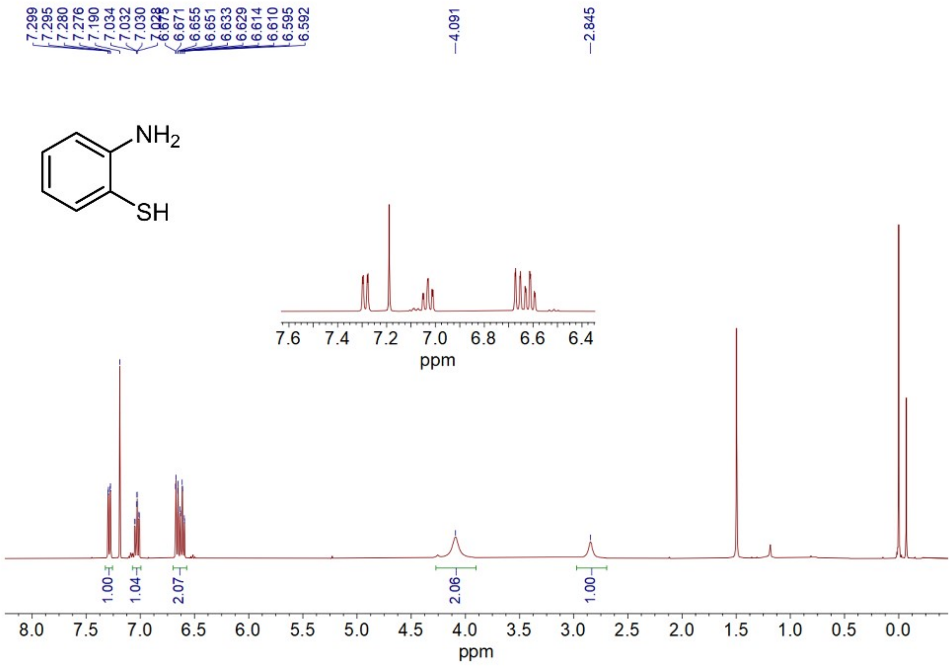

Figure S24. ^{1}H NMR spectrum of **M5** in $CDCl_3$.

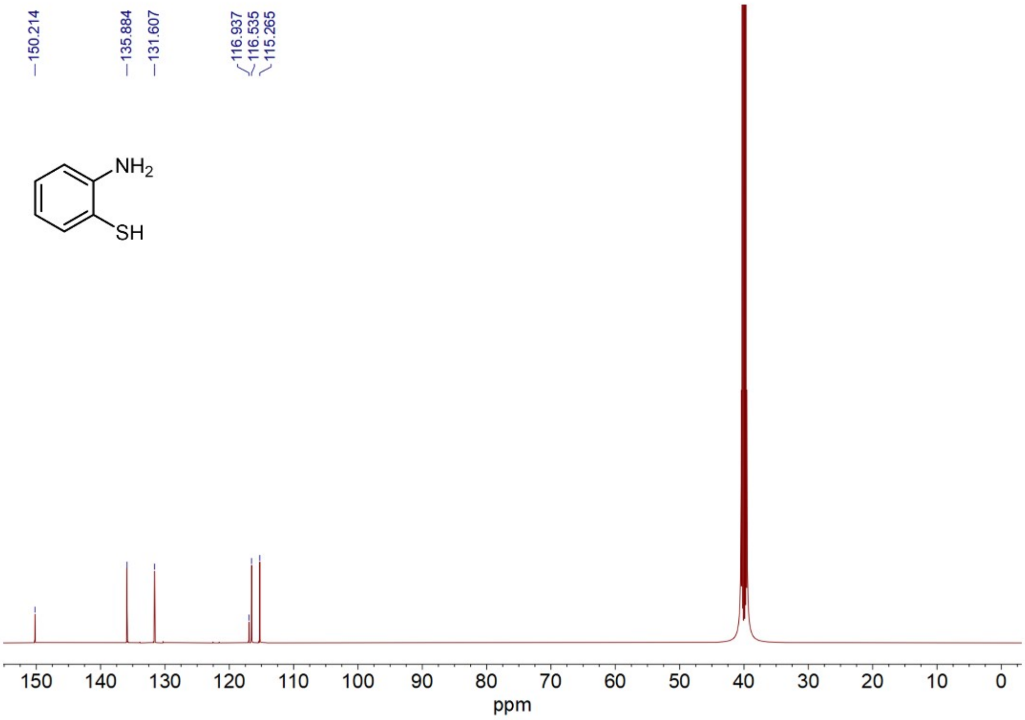

Figure S25. ^{13}C NMR spectrum of **M5** in DMSO-d_6.

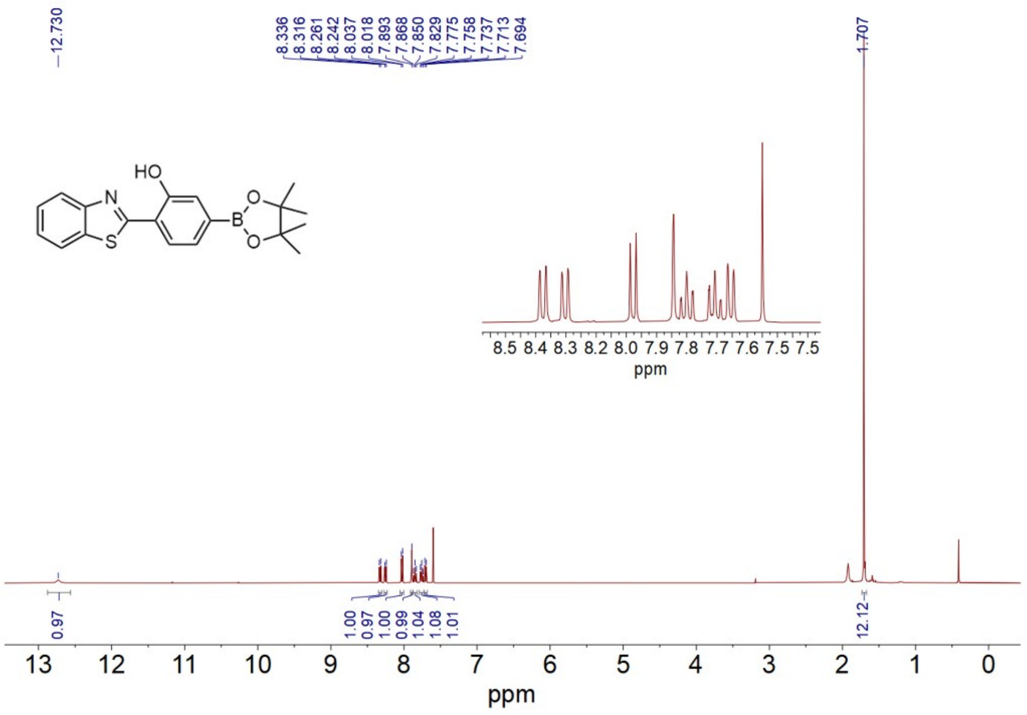

Figure S26. ^{1}H NMR spectrum of **M8** in $CDCl_3$.

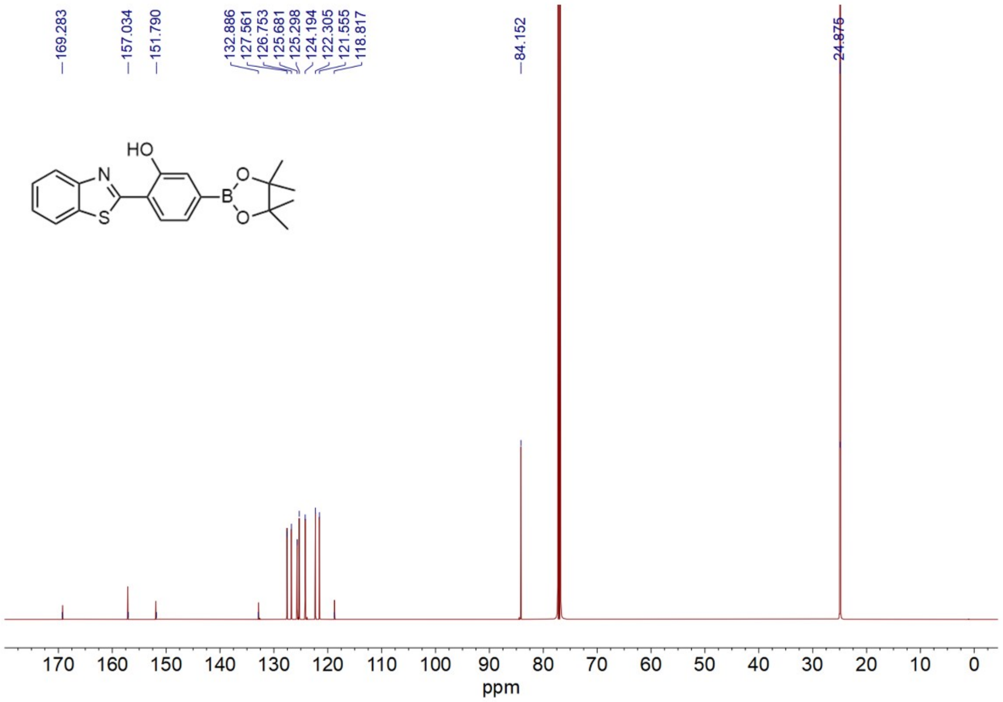

Figure S27. ^{13}C NMR spectrum of **M8** in $CDCl_3$.

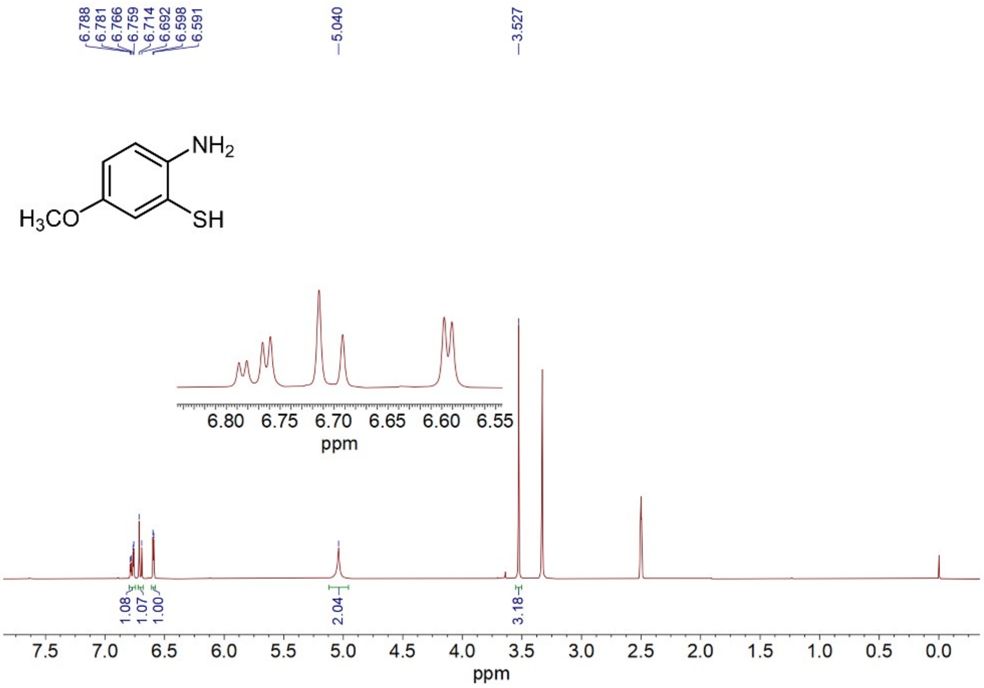

Figure S28. ^{1}H NMR spectrum of **M6** in DMSO-d_6

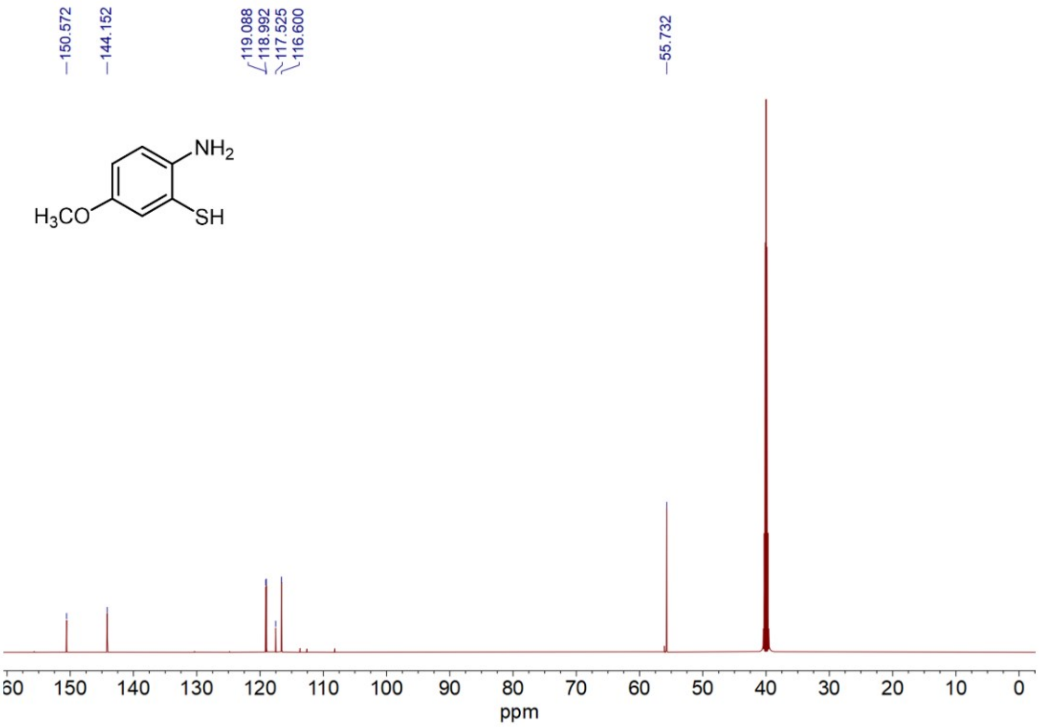

Figure S29. ^{13}C NMR spectrum of **M6** in DMSO-$d_{6.}$

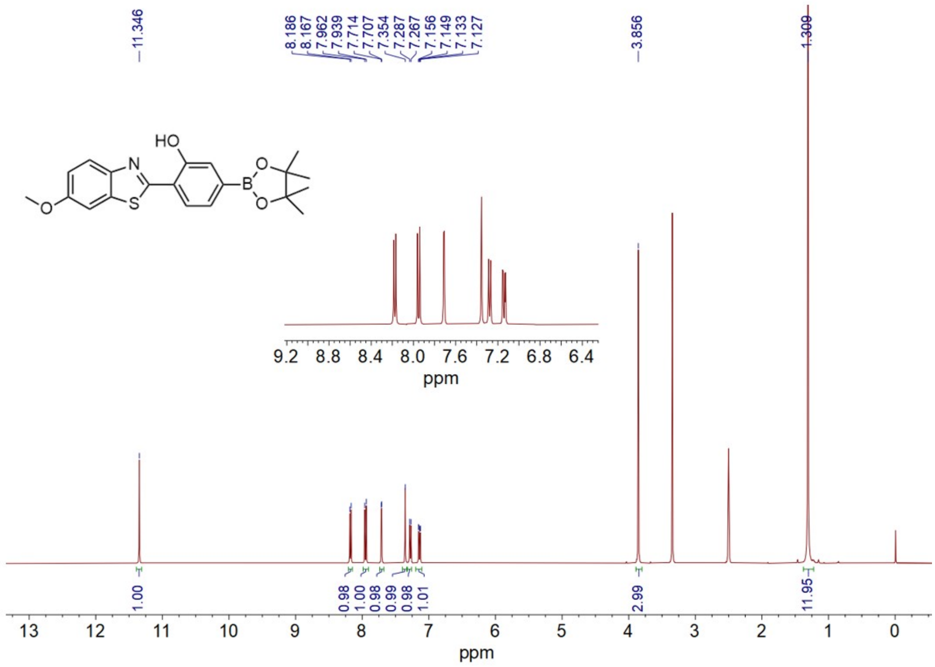

Figure S30. ^{1}H NMR spectrum of **M9** in $CDCl_3$

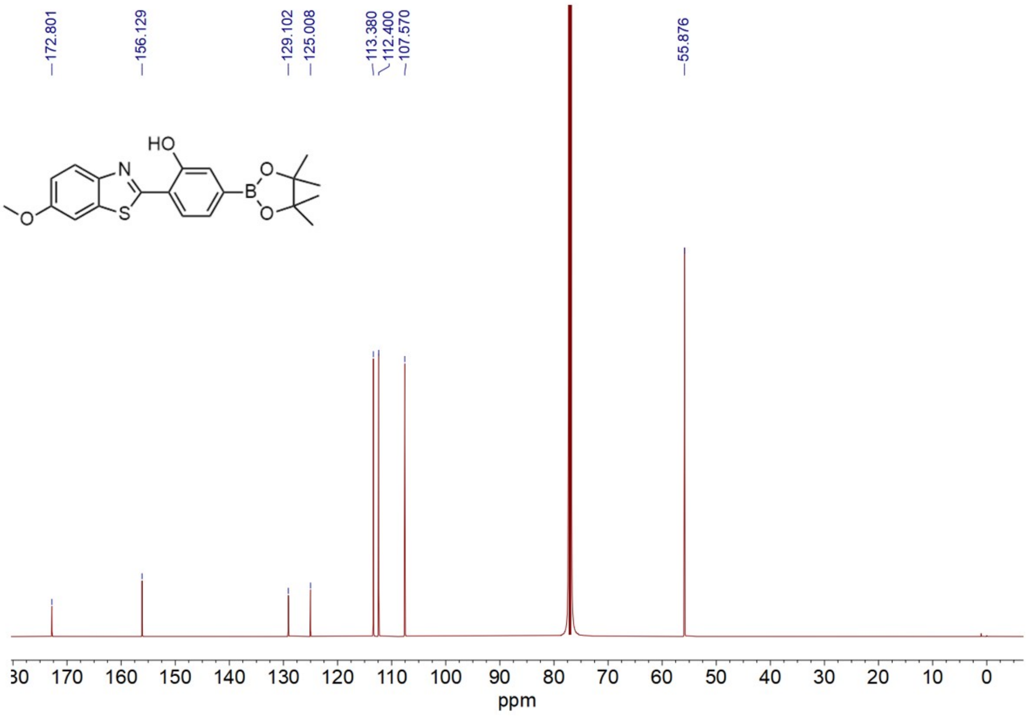

Figure S31. ^{13}C NMR spectrum of **M9** in $CDCl_3$.

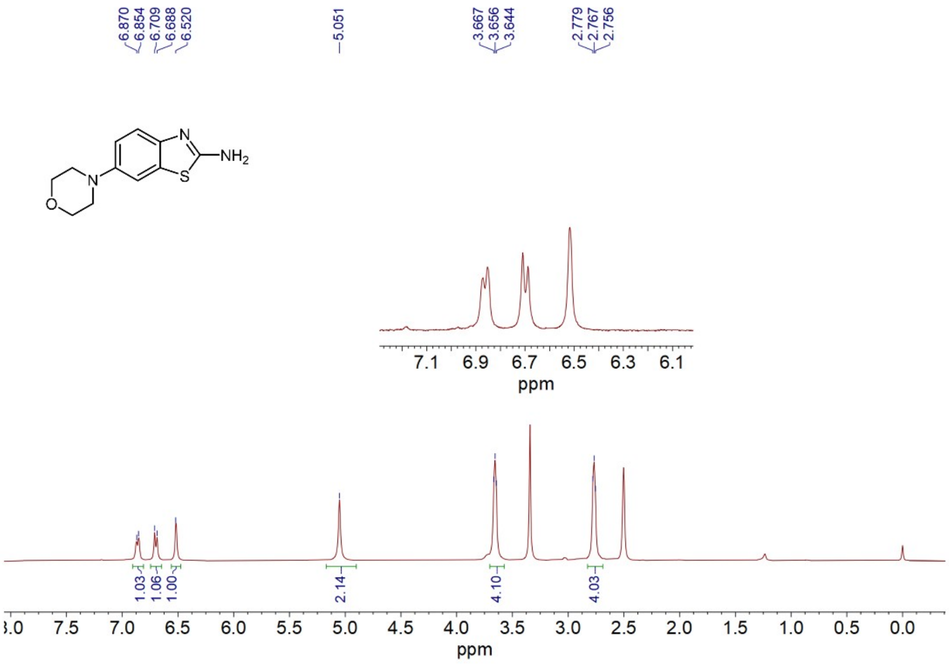

Figure S32. ^{1}H NMR spectrum of **M2** in DMSO-d_6

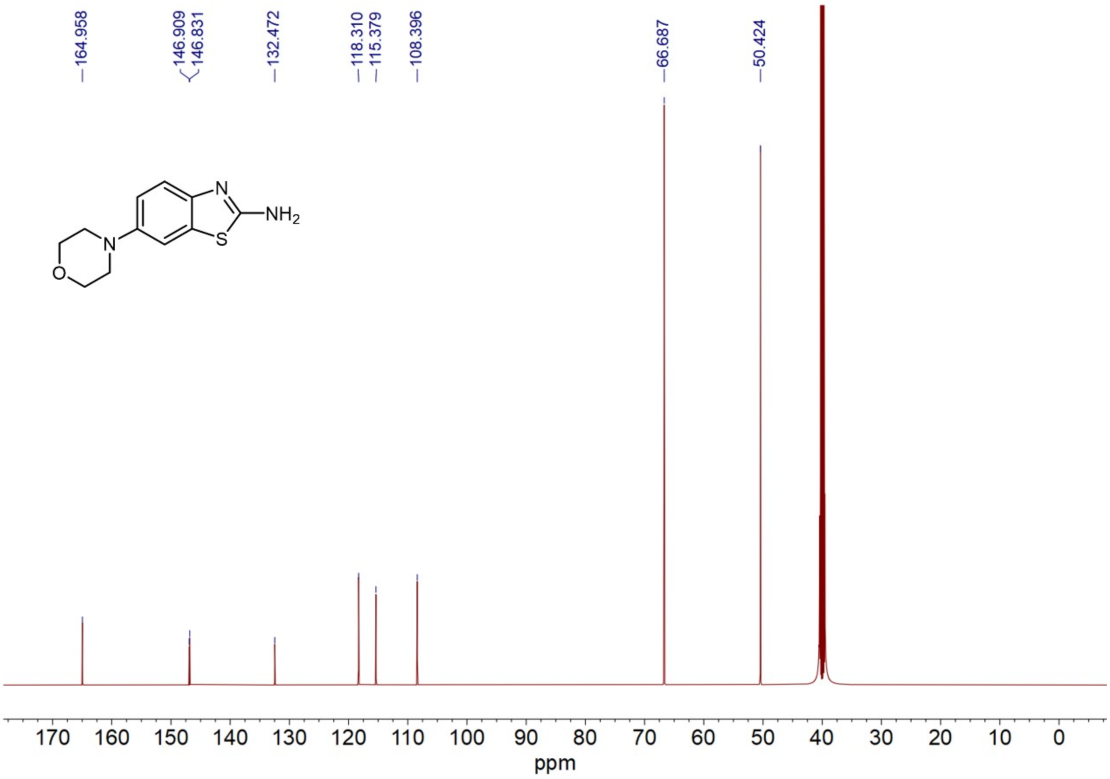

Figure S33. ^{13}C NMR spectrum of **M2** in DMSO-d_6.

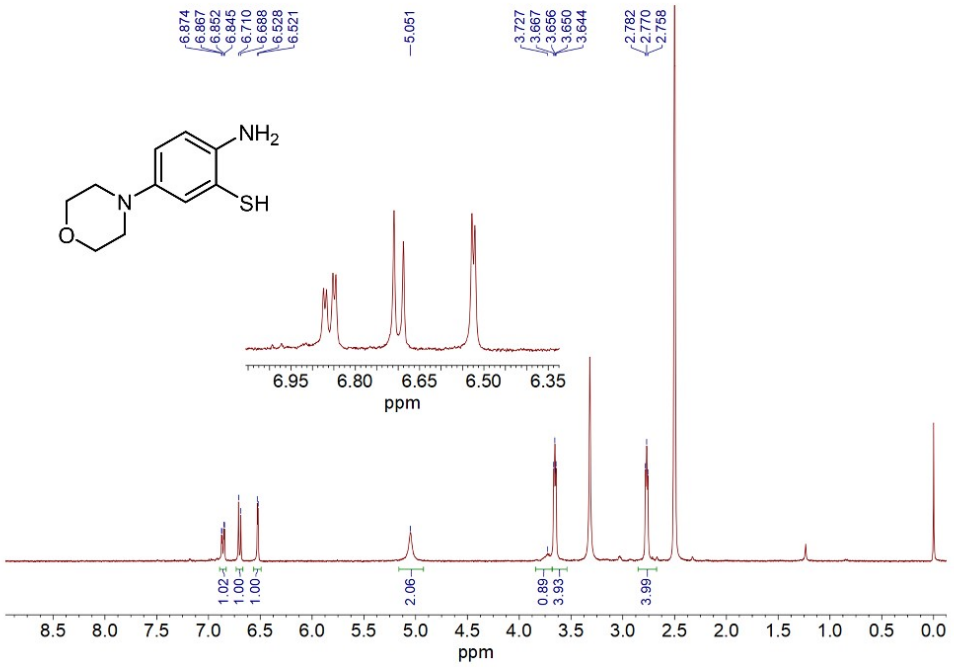

Figure S34. ^{1}H NMR spectrum of **M7** in DMSO-d_6

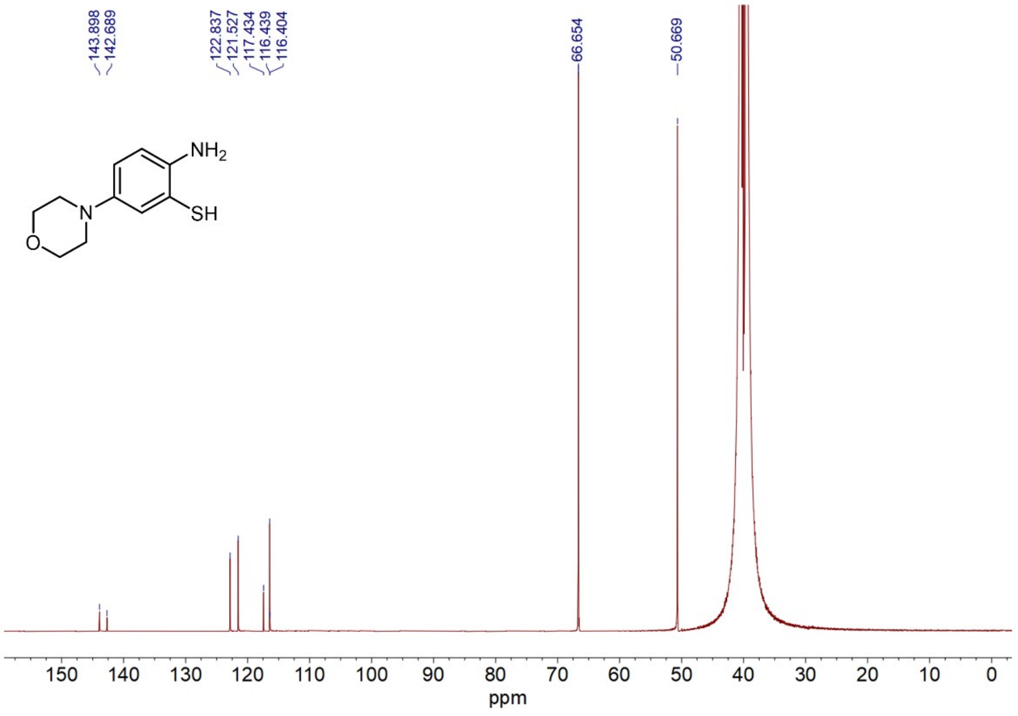

Figure S35. ^{13}C NMR spectrum of **M7** in DMSO-d_6.

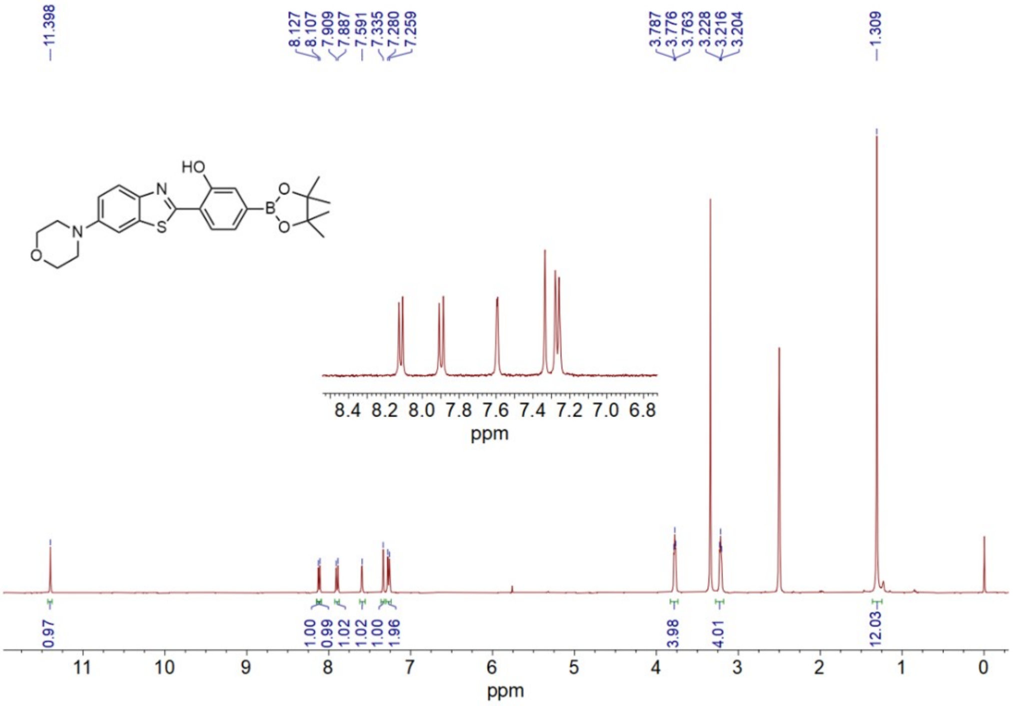

Figure S36. ^{1}H NMR spectrum of **M10** in DMSO-d_6

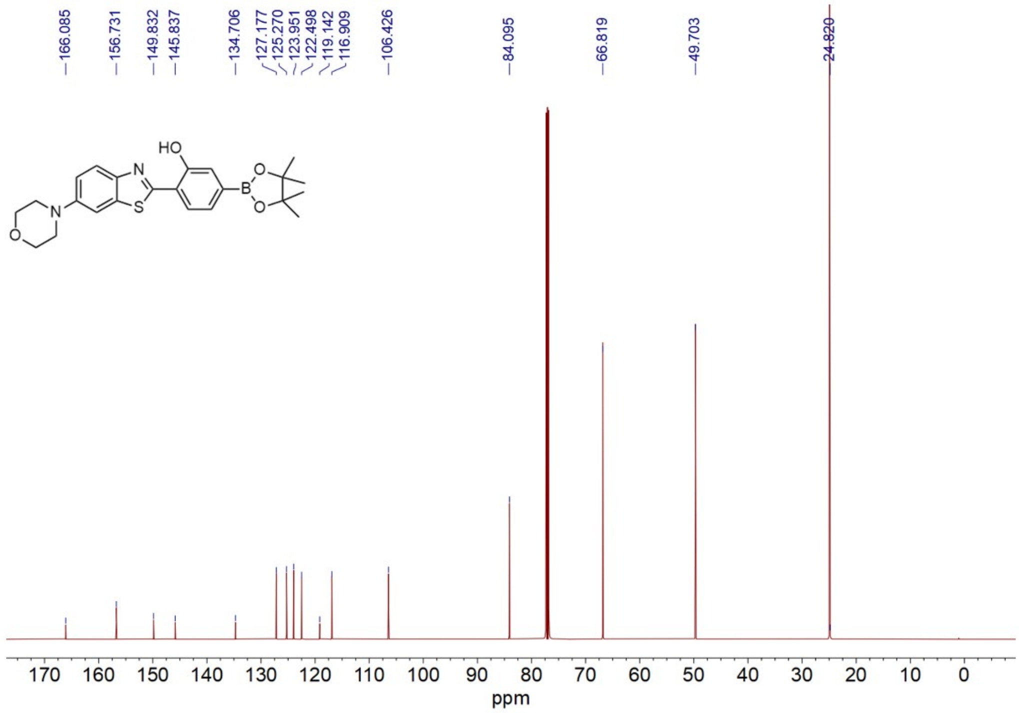

Figure S37. ^{13}C NMR spectrum of **M10** in $CDCl_3$.

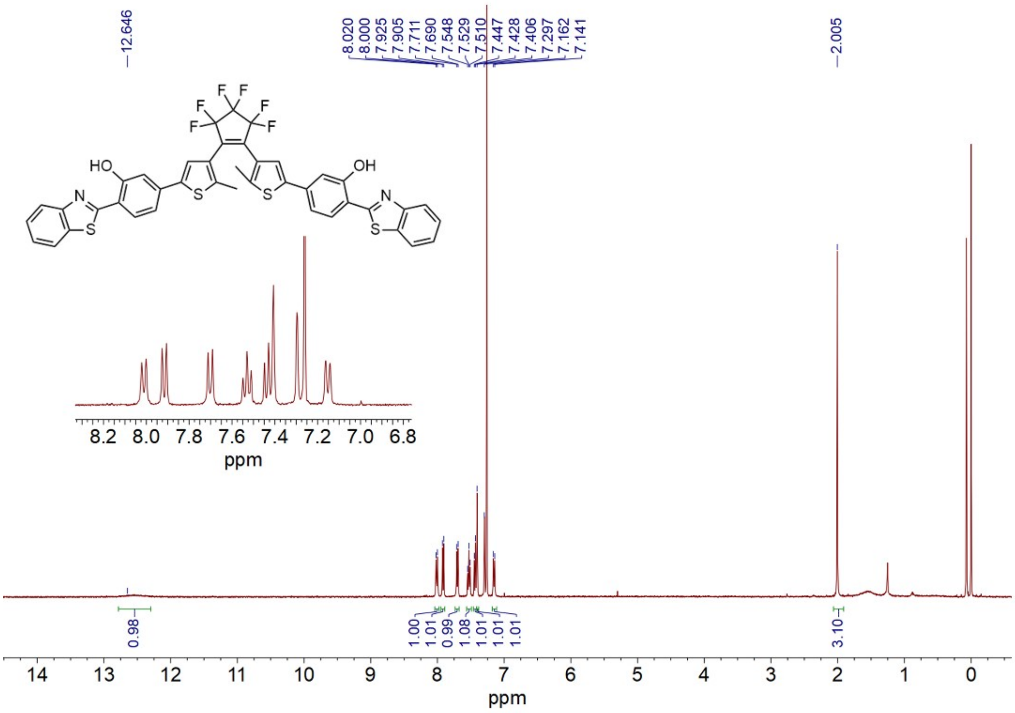

Figure S38. ^{1}H NMR spectrum of **DH** in $CDCl_3$

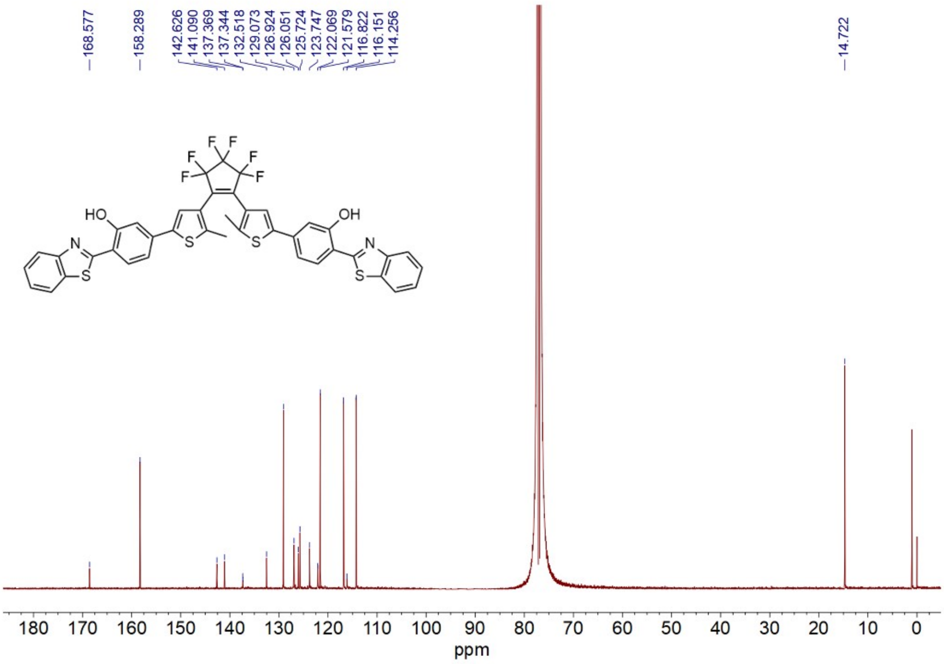

Figure S39. ^{13}C NMR spectrum of **DH** in $CDCl_3$

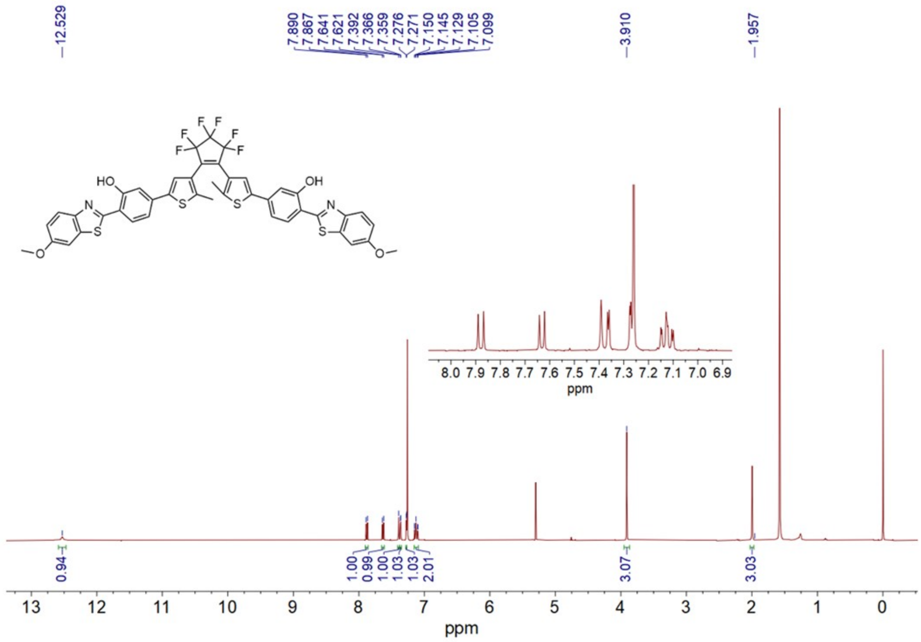

Figure S40. ^{1}H NMR spectrum of **DH-OMe** in $CDCl_3$

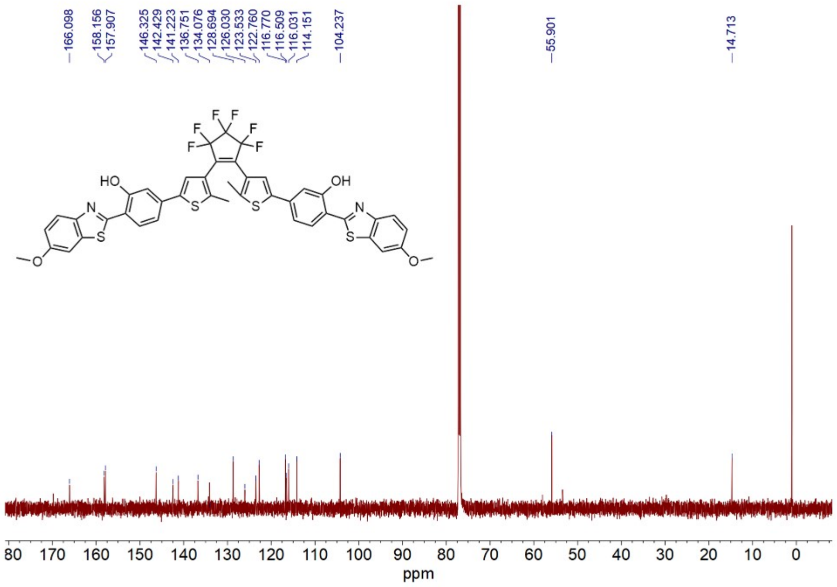

Figure S41. ^{13}C NMR spectrum of **DH-OMe** in $CDCl_3$

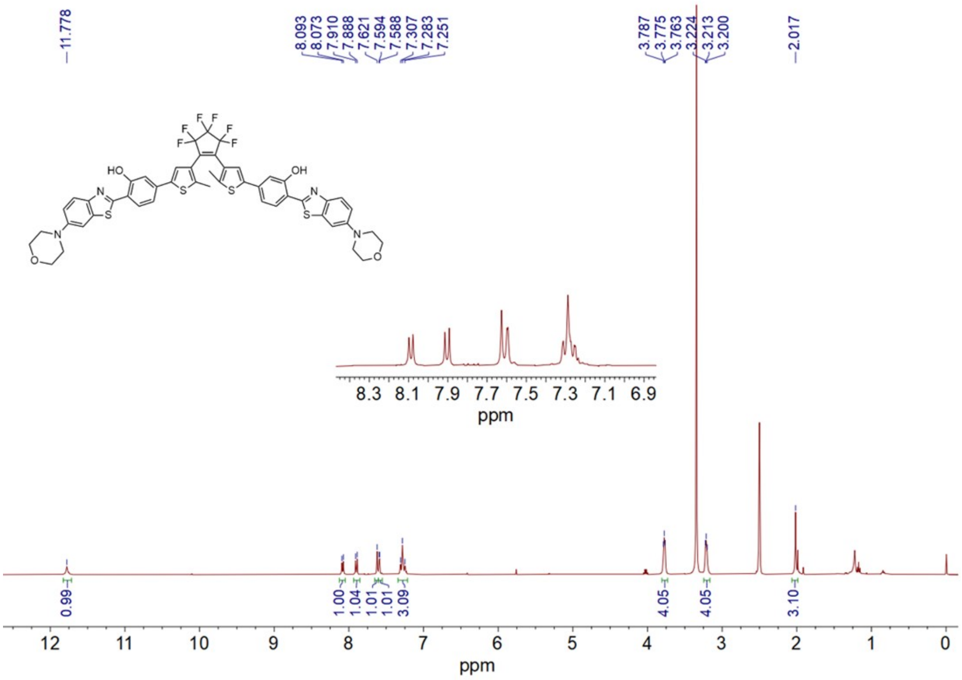

Figure S42. ^{1}H NMR spectrum of **DH-Mor** in DMSO-d_6

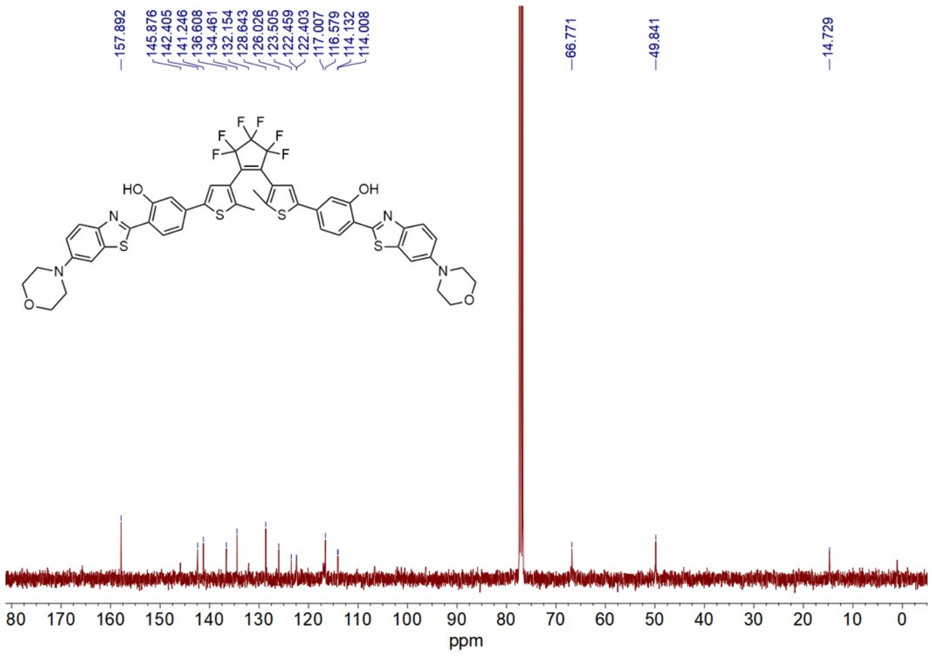

Figure S43. ^{13}C NMR spectrum of **DH-Mor** in $CDCl_3$

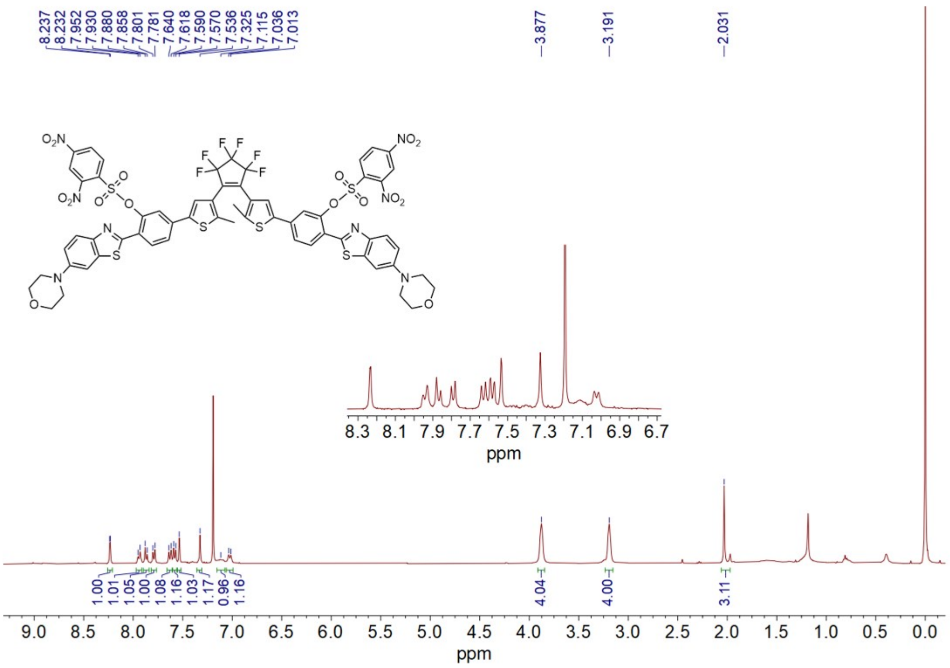

Figure S44. ^{1}H NMR spectrum of **DH-Mor-GSH** in $CDCl_3$

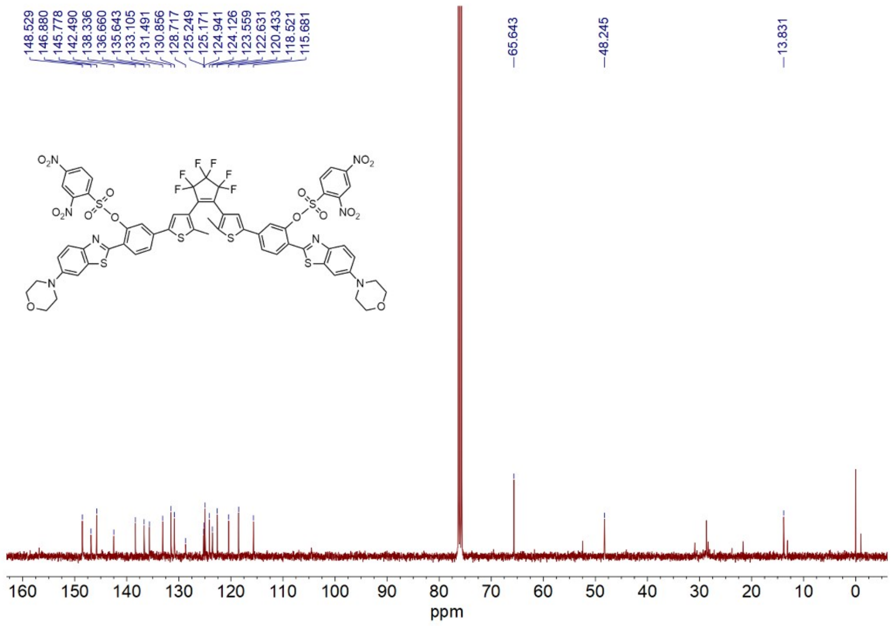

Figure S45. ^{13}C NMR spectrum of **DH-Mor-GSH** in $CDCl_3$

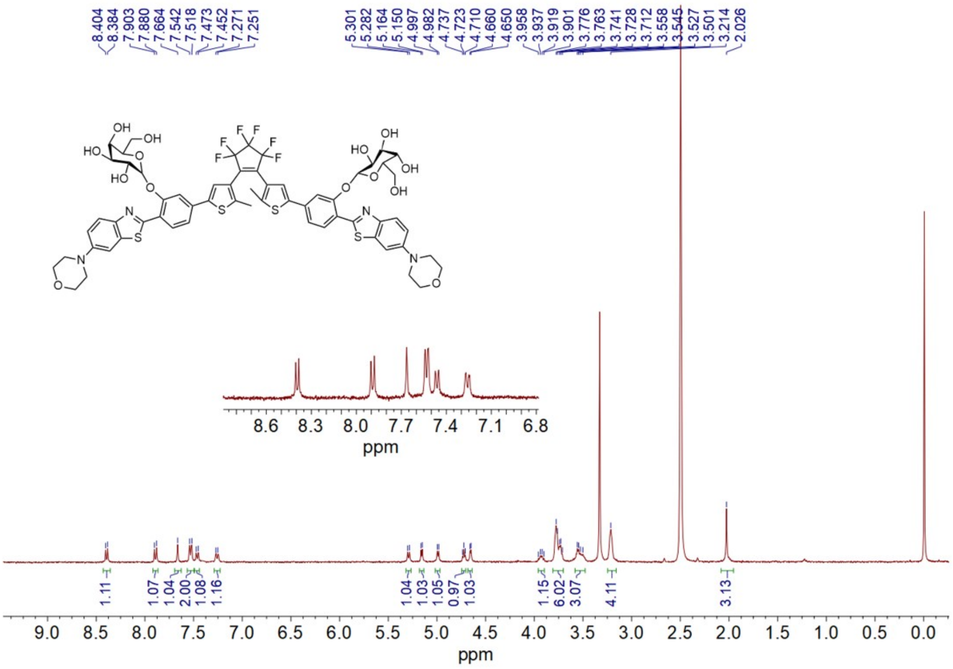

Figure S46. ^{1}H NMR spectrum of **DH-Mor-*β*-Gal** in DMSO-d_6

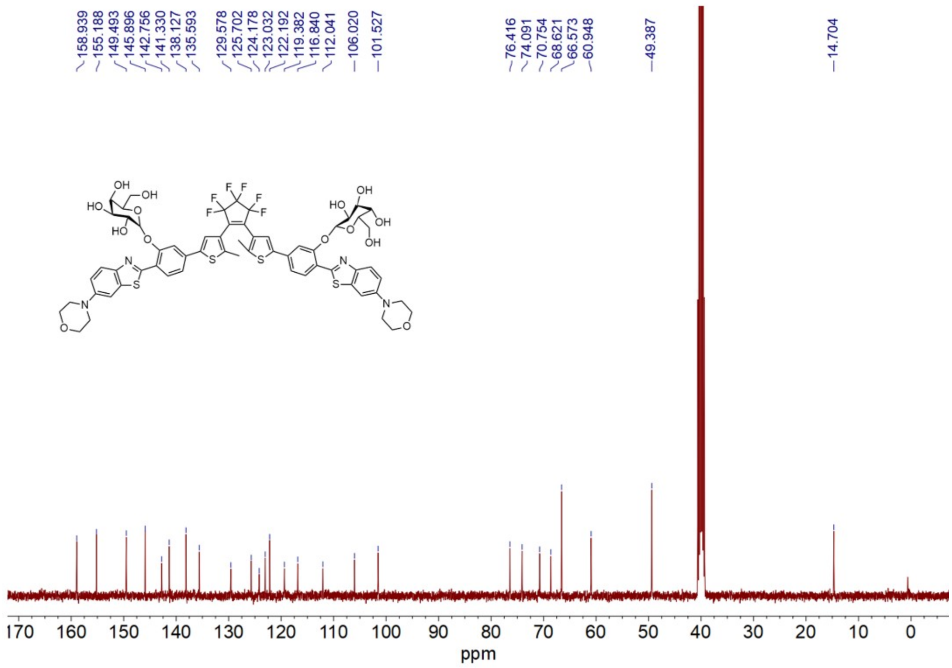

Figure S47. ^{13}C NMR spectrum of **DH-Mor-*β*-Gal** in DMSO-d_6

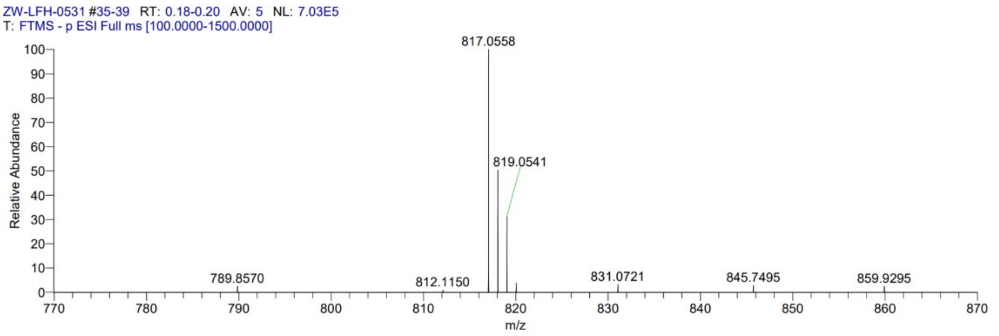

ZW-LFH-0531#35-39 RT: 0.18-0.20 AV: 5
T: FTMS - p ESI Full ms [100.0000-1500.0000]
m/z= 815.97-820.30

m/z	Intensity	Relative	Theo. Mass	Delta (ppm)	Composition
817.0558	711327.8	100.00	817.0562	-0.42	$C_{39}H_{24}ON_4F_5KNaS_3$

Figure S48. Mass spectrum of **DH**

Monoisotopic Mass, Even Electron Ions
489 formula(e) evaluated with 1 results within limits (up to 50 best isotopic matches for each mass)
Elements Used:
C: 0-43 H: 0-27 N: 0-2 O: 0-4 F: 0-6 S: 0-4
WH-ZHU
ZW-LFH-IPT 188 (4.147)

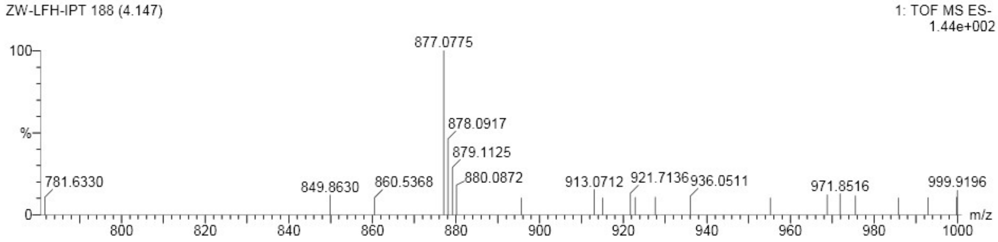

Minimum:				-1.5			
Maximum:		5.0	5.0	50.0			
Mass	Calc. Mass	mDa	PPM	DBE	i-FIT	i-FIT (Norm)	Formula
877.0775	877.0758	1.7	1.9	28.5	13.9	0.0	C43 H27 N2 O4 F6 S4

Figure S49. Mass spectrum of **DH-OMe**

Monoisotopic Mass, Even Electron Ions
1122 formula(e) evaluated with 1 results within limits (up to 50 best isotopic matches for each mass)
Elements Used:
C: 0-49 H: 0-40 N: 0-4 O: 0-6 F: 0-6 S: 0-4
WH-ZHU
ZW-LFH-2 6 (0.056) Cm (6)

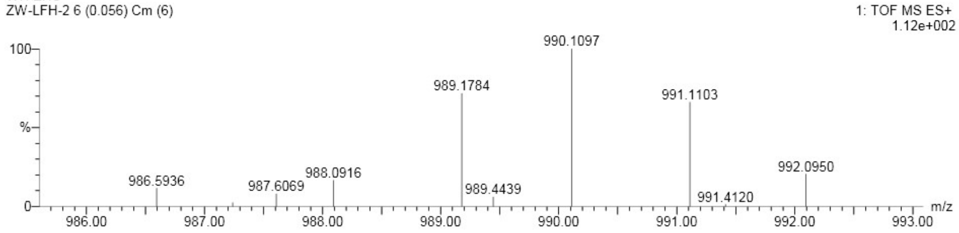

Minimum:				-1.5			
Maximum:		5.0	10.0	50.0			
Mass	Calc. Mass	mDa	PPM	DBE	i-FIT	i-FIT (Norm)	Formula
989.1784	989.1758	2.6	2.6	29.5	28.7	0.0	C49 H39 N4 O4 F6 S4

Figure S50. Mass spectrum of **DH-Mor**

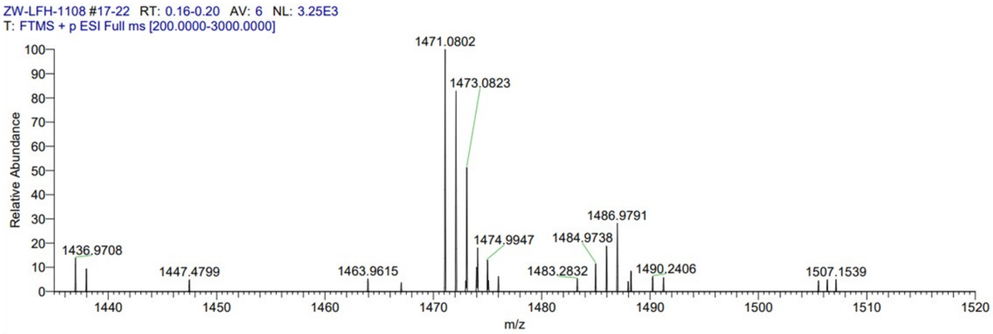

ZW-LFH-1108#17-22 RT: 0.16-0.20 AV: 6
T: FTMS + p ESI Full ms [200.0000-3000.0000]
m/z= 1470.91-1471.24

m/z	Intensity	Relative	Theo. Mass	Delta (ppm)	Composition
1471.0802	3260.0	100.00	1471.0839	-3.70	$C_{61}H_{42}O_{16}N_8F_6NaS_6$

Figure S51. Mass spectrum of **DH-Mor-GSH**

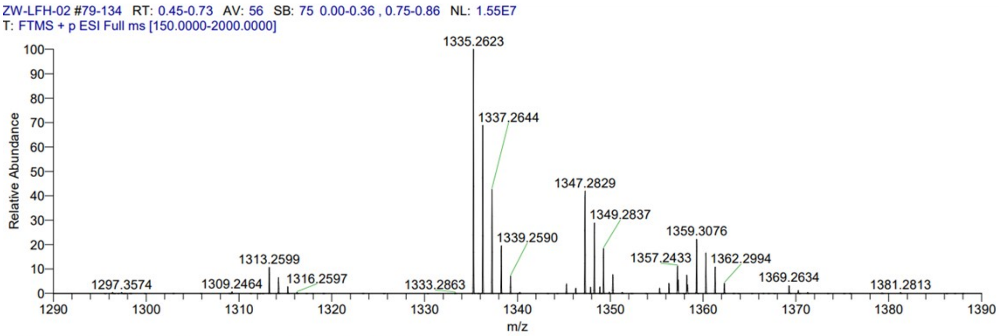

ZW-LFH-02#78-134 RT: 0.44-0.73 AV: 57
T: FTMS + p ESI Full ms [150.0000-2000.0000]
m/z= 1334.90-1335.48

m/z	Intensity	Relative	Theo. Mass	Delta (ppm)	Composition
1335.2623	15304095.0	100.00	1335.2629	-0.55	$C_{61}H_{58}O_{14}N_4F_6NaS_4$

Figure S52. Mass spectrum of **DH-Mor-*β*-Gal**

4. References

[1] Li C, Xiong K, Chen Y et al. *ACS Appl Mater Interfaces,* 2020, **12**, 27651-27662.

[2] A. T. R. Williams, S. A. Winfield, J. N. Miller. *Analyst*, 1983, **108**, 1067.

[3] H.J. Yvon, A Guide To Recording Fluorescence Quantum Yields, HORIBA Jobin Yvon Inc., Middlesex, UK, 2012.

[4] A. Ribeiro, R. Ballardini, P. Belser, M. T. Gandolfi, V. M. Iyer, L. Moggi. *Photochem. Photobiol. Sci*., 2009, **8**, 1734.

[5] Handbook of Photochemistry [M]. 3rd ed. Boca Raton, FL, USA: CRC press, 2006, 601-611

[6]. Li M, Hu H, Liu B et al. *J Am Chem Soc*., 2022, **144**, 20773-20784.
